# Mechanistic mass transport analysis of dissolution of weak-base drugs considering the influence of surface pH in formulations with and without acidifying agents

**DOI:** 10.3389/fphar.2026.1924679

**Published:** 2026-10-01

**Authors:** Venkata Krishna Kowthavarapu, Nitin Bharat Charbe, Tatiana Iakovleva, Cordula Stillhart, Neil John Parrott, Stephan Schmidt, Rodrigo Cristofoletti

**Affiliations:** 1 Department of Pharmaceutics, Center for Pharmacometrics and Systems Pharmacology, College of Pharmacy, University of Florida, Orlando, FL, United States; 2 Pharmaceutical Research & Development, Formulation & Process Development, F. Hoffmann—La Roche Ltd., Basel, Switzerland; 3 Pharmaceutical Research & Early Development, Roche Innovation Center Basel, F. Hoffmann—La Roche Ltd., Basel, Switzerland

**Keywords:** acidifying agents, diffusion, dissolution modeling, mass transport analysis, surface pH

## Abstract

At the solid–liquid interface, the dissolution of weakly basic drugs, particularly those exhibiting poor solubility, is highly sensitive to local pH conditions. These compounds frequently show pH-dependent solubility and reduced bioavailability when co-administered with acid-reducing agents, which presents significant difficulties for formulation design and clinical efficacy. Despite advances in physiologically based biopharmaceutics modeling, most existing dissolution modeling frameworks fail to account for microenvironmental pH changes induced by functional excipients such as acidifying or basifying agents. To address this critical gap, we developed a mechanistic mass-transport-based dissolution framework that explicitly quantifies surface pH as a function of excipient diffusion and acid–base equilibria. The model integrates coupled diffusion–reaction kinetics within the diffusion boundary layer, enabling accurate prediction of interfacial pH under both buffered and unbuffered conditions. Building on the classical framework, we numerically captured the impact of monoprotic and diprotic acidifying agents on surface pH. Model validation was performed using entrectinib, a clinically relevant weak base, across formulations with and without acidifying agents, supported by experimental dissolution data generated under different media conditions. The results demonstrate that bulk pH-based modeling is insufficient to capture critical formulation-driven dissolution behavior and establish surface pH as a primary determinant of dissolution kinetics. Overall, this study establishes surface pH modeling as a key advancement for mechanistic dissolution prediction of oral drug products containing functional pH-modifying excipients.

## Graphical Abstract

**Figure:**
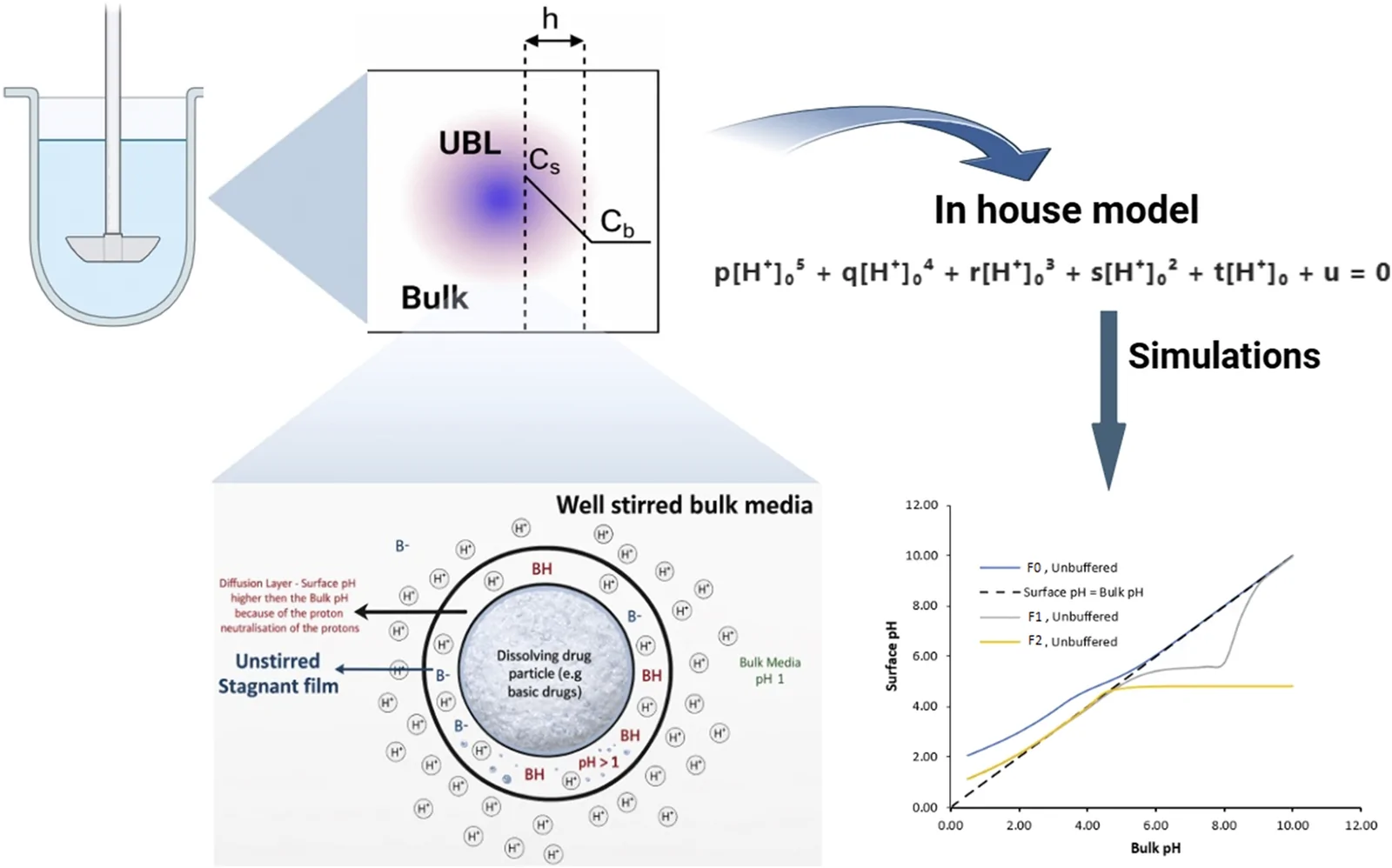

## 1 Introduction

Computational modeling and simulation of oral drug absorption in the gastrointestinal (GI) tract have been utilized to mechanistically understand and quantify the interaction of drug product quality attributes influencing *in vivo* drug performance, which may significantly impact the drug development and approval process (Tsume et al., 2018; Mooney et al., 1981a; Mooney et al., 1981b). Despite the availability of various computational models to characterize the dissolution of oral drug products in the GI tract, these models have limited capabilities to predict the formulation effect on drug dissolution (Loisios-Konstantinidis and Dressman, 2020; Heimbach et al., 2024; Wu and Li, 2023). Traditionally, efforts to enhance oral absorption models have primarily focused on incorporating the physicochemical properties of the active pharmaceutical ingredient (API), such as pKa, solubility, particle size, crystal form, and drug diffusivity (Heimbach et al., 2024; Wu and Li, 2023; Amidon et al., 1995; Shah et al., 2014). However, the dissolution of API can also be influenced by fluid buffer composition and formulation excipients that can modulate the pH at the solid–liquid interface of dissolving particles (Mooney et al., 1981a; Mooney et al., 1981b). Especially, if we have an ionizable drug, an acid, or a base, we must consider the reaction occurring at the surface of the solid particle. This was a concept introduced by Higuchi et al. (1958). If we have an acid base reaction occurring on the surface, protons are consumed or generated that may change the pH at the surface and thereby the solubility at the surface of the drug. Therefore, solubility at the drug particle surface is driving dissolution as repeatedly demonstrated by multiple authors (Mooney et al., 1981a; Mooney et al., 1981b; Ozturk et al., 1988; Salehi et al., 2021; Serajuddin and Jarowski, 1985a; Serajuddin and Jarowski, 1985b).

The model by Mooney et al. generated the theoretical framework for estimating the surface pH and described the dissolution kinetics of monoprotic carboxylic acids (e.g., benzoic acid, 2-naphthoic acid, and indomethacin) from a rotating compressed disk into aqueous solution in a USP II apparatus, focusing on pH-dependent dissolution processes under unbuffered and buffered conditions (Mooney et al., 1981a; Mooney et al., 1981b). Rooted in boundary layer theory, the model integrates diffusion-controlled mass transport with ionization reactions (e.g., HA ⇌ H^+^ + A^−^) within a thin aqueous diffusion layer at the solid–liquid interface. Unlike the stagnant film model, it treats mass transfer as a diffusion process, incorporates heterogeneous acid–base reactions, and uses experimental diffusion coefficients for all species (including hydronium ion [H^+^] and hydroxide ion [OH^−^]). The model assumes instantaneous ionization equilibrium and constant initial steady-state flux, leveraging Fick’s second law to predict concentration profiles and dissolution rates (Pepin et al., 2019).

An important feature of this model is the surface pH at the solid–liquid interface. For example, the surface pH for benzoic acid in an unbuffered aqueous solution at pH 7 is significantly more acidic (i.e., pH 3–4) than the pH in the surrounding medium, referred to as bulk solution pH (i.e., pH 7), due to the release of H^+^ ions during acid dissolution. In contrast, buffer species (e.g., B and BH^+^) counteract the acidic microclimate by neutralizing H^+^ ions through chemical equilibria in buffered systems (Mooney et al., 1981a). The model by Mooney et al. employs intrinsic physicochemical parameters, such as solubility, pKa, and diffusion coefficients, to accurately predict dissolution rates and surface pH. The diffusion coefficient of the drug is estimated from the Stokes–Einstein equation, which correlates the diffusion coefficient of a drug particle to its density and the viscosity of medium (Kowthavarapu et al., 2024). By consolidating complex buffer equilibria into a single flux boundary condition, it simplifies the mathematical framework. The model was further validated against the experimental data (Mooney et al., 1981a; Mooney et al., 1981b); it quantifies the ionized species contribution to total flux as a function of bulk pH, making it a valuable tool for improving the prediction of oral drug absorption.

However, the Mooney model focuses on the intrinsic drug properties such as pKa and solubility to predict dissolution kinetics and is constrained by its limited consideration of pH-modifying excipients (e.g., succinic acid and magnesium oxide). These excipients actively modulate the surface pH microclimate beyond the contributions of the drug or conventional buffer systems, thereby influencing ionization and dissolution dynamics (Pepin et al., 2019; Pepin et al., 2024). Conversely, basifying excipients, such as magnesium oxide, elevate the surface pH, promoting ionization and enhancing dissolution (Salehi et al., 2021; Badawy and Hussain, 2007). For instance, basifying agents are employed in formulations such as esomeprazole, a weak acid, to create an alkaline microenvironment that mitigates gastric acid degradation and augments dissolution (Van Nguyen et al., 2017). Similarly, acidifying excipients enhance the dissolution and bioavailability of weak base drugs such as famotidine by acidifying the microenvironment, thereby increasing their solubility (Mitra and Kesisoglou, 2013). The present Mooney model framework does not account for the diffusion or chemical equilibria of these excipient-driven reactions, limiting its applicability to complex pharmaceutical formulations designed to optimize dissolution and bioavailability.

To overcome this limitation of the Mooney model, additional species (excipient molecules, ionized forms, and reaction products) and their equilibria (e.g., acid dissociation) must be incorporated. This requires solving coupled diffusion–reaction equations to capture the modified pH profiles and surface pH, followed by validation with experimental data. Such an extension holds great promise for enhancing the model utility for pharmaceutical development, enabling precise prediction and control of dissolution kinetics through strategic excipient selection, particularly for poorly soluble drugs.

The important step in accurately determining the dissolution rate of drug products containing a pH-modifying excipient (acidifying/basifying agent) is to estimate the particle surface pH at the dissolving surface. The surface pH plays a key role in influencing drug solubility at the solid–liquid interface and is primarily dependent on the concentration of [H+] present in this microenvironment. This local [H+] concentration is governed by both the dissociation constant and concentrations of the ionizable species near the particle surface, making it crucial to model the chemical equilibria accurately (Salehi et al., 2021). Diffusivity is another significant factor that impacts the concentrations of these species at the dissolving particle surface. A species with faster diffusion will reduce its concentration at the dissolving particle surface (Serajuddin and Jarowski, 1985a). Specifically, the difference in diffusivities between the drug and the pH-modifying excipient affects how their respective concentrations evolve at the interface of the diffusion layer. A species with a higher diffusivity will more rapidly move away from the dissolving surface, thereby reducing its local concentration and altering the surface pH. This becomes particularly important in drug formulations that include strong acidifying or basifying agents, which interact with the drug at the interface and modulate its local solubility (Salehi et al., 2021). These excipients are often highly water-soluble and exhibit significantly higher aqueous solubility than the intrinsic solubility of the API. Therefore, it was assumed that the dissolution of the pH-modifying excipient is not the rate-limiting step, with its species being dissolved in the bulk medium from the starting of the dissolution process (Salehi et al., 2021; Serajuddin and Jarowski, 1985a; Serajuddin and Jarowski, 1985b).

The mass transport analysis was performed using Fick’s law of diffusion to describe the dissolution process by incorporating simultaneous chemical reactions taking place in the solid–liquid boundary layer adjacent to the dissolving drug particle surface to predict the surface pH (Mooney et al., 1981a; Mooney et al., 1981b; Higuchi et al., 1958; Ozturk et al., 1988; Serajuddin and Jarowski, 1985a; Serajuddin and Jarowski, 1985b). The aim of this study is to develop a modeling framework for the prediction of surface pH and to evaluate the effect of the type and the amount of the acidifying agent on the dissolution rate of a weakly basic drug, using entrectinib as a prototypical model compound (Parrott et al., 2020). Entrectinib, a potent and selective tyrosine kinase inhibitor, is approved for the treatment of ROS1 fusion-positive non-small cell lung cancer and NTRK fusion-positive solid tumors (Parrott et al., 2020). Specifically, we constructed analytical solutions for four distinct scenarios: a dibasic active interacting with Equation 1 with no acidifier, a monoprotic acidifier (Mooney et al., 1981b), and a diprotic acidifier under unbuffered conditions (Tsume et al., 2018), as well as these combinations under buffered conditions (Loisios-Konstantinidis and Dressman, 2020). We choose to derive explicit analytical polynomial solutions, rather than relying purely on numerical solvers for arbitrary mixtures to establish transparent, mechanistic mathematical algorithms that directly demonstrate the interplay of individual diffusivities and acid–base equilibria. However, for broader formulations featuring complex, arbitrary selections of pH-modifying agents, numerical solvers for simultaneous equilibria can be readily implemented based on the fundamental principles described in this manuscript.

## 2 Materials and methods

To develop effective quantitative models, it is essential to integrate the physicochemical properties that influence drug dissolution both *in vitro* and *in vivo*, to account for physiological variability. Surface pH, also known as microclimate pH, plays a key role in determining the dissolution rate of an ionizable drug. The concentration of [H+] ions at the solid–liquid interface may differ from that in the bulk medium. Accordingly, the solubility at the solid surface (Ssurface) is not equivalent to the solubility of the drug in the bulk medium (Mooney et al., 1981a; Mooney et al., 1981b; Pepin et al., 2019). Therefore, it is important to consider the effect of surface pH to account for the self-buffering property of a free base drug and to understand the influence of a pH-modifying excipient (acidifying agent).

The mass transport analysis applies boundary layer theory, which proposes that an unstirred diffusion boundary layer exists in the immediate vicinity of the drug particle’s dissolving surface (Higuchi et al., 1958; Uekusa et al., 2022). The dissolution process is controlled by the concentration gradient across the boundary layer and is affected by the simultaneous diffusion and chemical reactions occurring at the dissolving surface and within the boundary layer (Mooney et al., 1981a; Mooney et al., 1981b; Ozturk et al., 1988). For the dissolution of a weak base with an acidifying excipient, the acidulant first dissolves according to its solubility product, which increases the concentration of hydronium ions [H+] and alters the surface pH within the diffusion layer. This change can significantly enhance the drug’s solubility, depending on the physiochemical property of the selected acidifying excipient (Salehi et al., 2021; Casalini et al., 2023).

A model was developed to predict the surface pH of a basic drug with a monoprotic or diprotic acidifying agent under both unbuffered and buffered conditions. In our model, we assumed that the aqueous solubility of the acidifying agents was significantly higher than the drug’s intrinsic solubility. Therefore, it was expected that the acidifying agent’s dissolution is not the rate-limiting step, and its molecular species are already dissolved in the bulk medium at the onset of the dissolution process (Salehi et al., 2021). The pH at the solid–liquid interface of the drug was calculated using charge and mass balance equations within the boundary layer (Mooney et al., 1981a; Mooney et al., 1981b; Uekusa et al., 2022).

The model assumes equilibrium reactions among all reactive species within the hydrodynamic boundary layer, predicting the surface pH based on factors such as the bulk pH, buffer and acidulant concentrations and pKa values, the drug’s pKa, and its intrinsic solubility (Salehi et al., 2021). It is important to note that the pH at the dissolving surface of an ionizable compound is usually different from the bulk pH due to the self-buffering property of the compound. The surface pH is affected by bulk pH, buffer concentration, and the mass transport of various species within the diffusion layer, resulting in a different pH at the particle dissolving surface.

To further explore the influence of mass transport and reactive species on the surface pH, specific cases involving weak bases in unbuffered and buffered solutions can provide valuable insights into the mechanisms governing the dissolution behavior. For instance, the dissolution of a dibasic drug in an unbuffered medium is governed by the interplay between diffusion and chemical reactions within the boundary layer, particularly in the presence of varying [H^+^] ion concentrations. By examining these cases, the model can delineate how the intrinsic properties of the drug and the presence of acidifying excipients can alter local pH conditions at the drug particle surface, influencing solubility and the dissolution rate (Mooney et al., 1981a; Mooney et al., 1981b; Salehi et al., 2021). To conceptualize this, Figure 1 illustrates the one-dimensional boundary value problem, depicting the solid–liquid interface and the concentration gradient of the reacting species across the diffusion layer.

**FIGURE 1:**
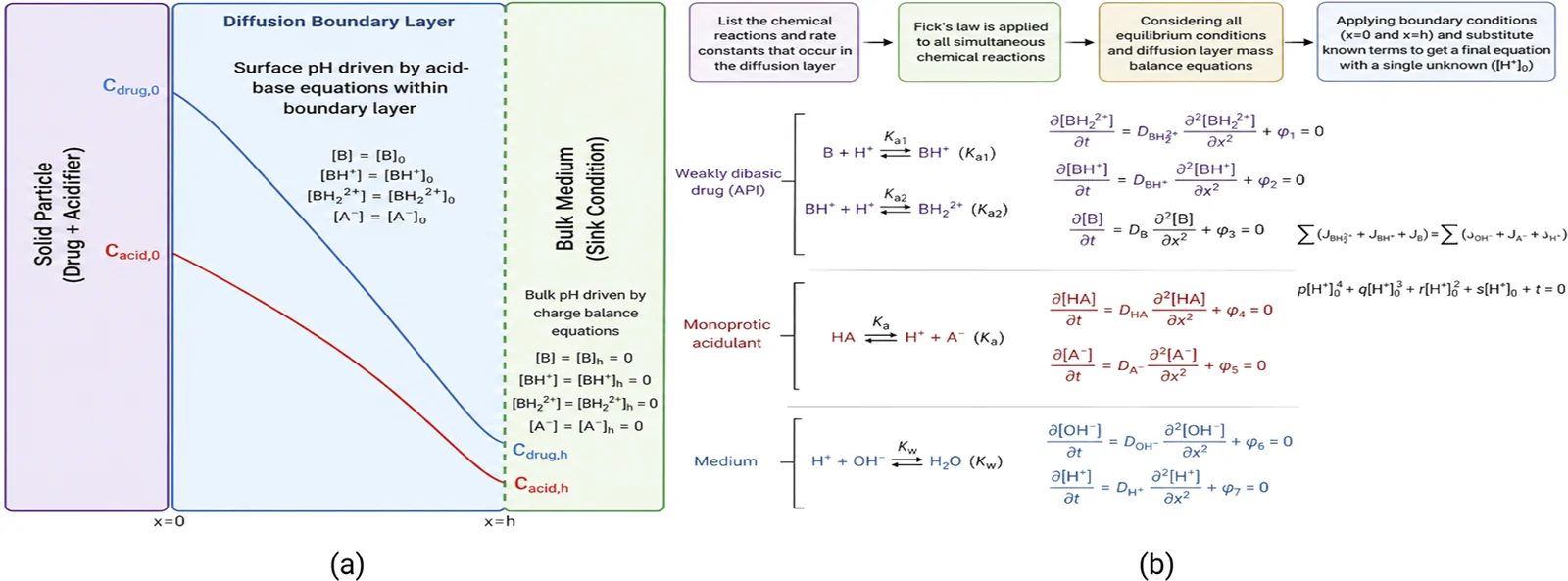
**(a)** Schematic representation of the 1-dimensional mass transport diffusion reaction model. It illustrates the solid–liquid interface (x = 0), the diffusion boundary layer thickness h, and the concentration gradients of the drug and acidifier species within the diffusion boundary layer. **(b)** Flow chart for surface pH calculation.

The flux of all species across the diffusion layer included both diffusion and chemical reactions occurring during dissolution. Using the simplified film model approach by Mooney et al., the differential equations that describe the diffusion and simultaneous chemical reactions of the individual species within the diffusion layer at steady state are defined as follows for a diprotic weak base with a monoprotic acidulant under unbuffered conditions (Kowthavarapu et al., 2024):

The chemical equilibria and reactions that take place when a dibasic drug, B, and a monoprotic acidulant, HA, dissolves under unbuffered conditions are presented in Equations 1–4:

$$B+H^{+}\overset{K_{a1}}{↔}BH^{+},$$ (1)

$$BH^{+}+H^{+}\overset{K_{a2}}{↔}BH_{2}^{2+},$$ (2)

$$HA\overset{K_{a3}}{↔}H^{+}+A^{-},$$ (3)

$$H^{+}+OH^{-}\overset{K_{w}}{↔}H_{2}O.$$ (4)

The chemical equilibrium rate constant reactions for the above reactions are presented in Equations 5–8:

$$K_{a1}=\frac{\left[B\right]\left[H^{+}\right]}{\left[BH^{+}\right]},$$ (5)

$$K_{a2}=\frac{\left[BH^{+}\right]\left[H^{+}\right]}{\left[BH_{2}^{2+}\right]},$$ (6)

$$K_{a3}=\frac{\left[H^{+}\right]\left[A^{-}\right]}{\left[HA\right]},$$ (7)

$$K_{w}=\left[H^{+}\right]\left[OH^{-}\right].$$ (8)

The parenthesis “[]” indicates the “dissolved concentration” of the molecular species. The flux of all species across the diffusion layer includes both diffusion and chemical reactions occurring during dissolution. Using the simplified film model approach by Mooney et al., the differential equations that describe the diffusion and simultaneous chemical reactions of the individual species within the diffusion layer at steady state are presented in Equations 9–15 (Mooney et al., 1981a; Mooney et al., 1981b):

$$\frac{\partial \left[BH_{2}^{2+}\right]}{\partial t}=D_{B}\frac{\partial^{2}\left[BH_{2}^{2+}\right]}{\partial x^{2}}+\phi_{1}=0,$$ (9)

$$\frac{\partial \left[BH^{+}\right]}{\partial t}=D_{B}\frac{\partial^{2}\left[BH^{+}\right]}{\partial x^{2}}+\phi_{2}=0,$$ (10)

$$\frac{\partial \left[B\right]}{\partial t}=D_{B}\frac{\partial^{2}\left[B\right]}{\partial x^{2}}+\phi_{3}=0,$$ (11)

$$\frac{\partial \left[\text{HA}\right]}{\partial t}=D_{\text{HA}}\frac{\partial^{2}\left[\text{HA}\right]}{\partial x^{2}}+\phi_{4}=0,$$ (12)

$$\frac{\partial \left[A^{-}\right]}{\partial t}=D_{A^{-}}\frac{\partial^{2}\left[A^{-}\right]}{\partial x^{2}}+\phi_{5}=0,$$ (13)

$$\frac{\partial \left[{\text{OH}}^{-}\right]}{\partial t}=D_{{\text{OH}}^{-}}\frac{\partial^{2}\left[{\text{OH}}^{-}\right]}{\partial x^{2}}+\phi_{6}=0,$$ (14)

$$\frac{\partial \left[H^{+}\right]}{\partial t}=D_{H^{+}}\frac{\partial^{2}\left[H^{+}\right]}{\partial x^{2}}+\phi_{7}=0.$$ (15)

Mass and charge balance considerations give the following relationships between $\phi_{i}$ terms, which are the reaction rate functions:

At equilibrium, the reaction rate of the reactant should be the opposite of the product. Based on the chemical equilibria, Equations 16, 17 can be obtained:

$$\phi_{1}=-\phi_{2}-\phi_{3},$$ (16)

$$\phi_{4}=-\phi_{5}-\phi_{7}.$$ (17)

The reaction rate of the acid and the base should be the same:

$$\phi_{1}+\phi_{4}+\phi_{7}=\phi_{3}+\phi_{5}+\phi_{6}.$$ (18)

This equation ensures that the net change in charge due to the reactions is zero.

Based on Equations 16–18, the following mass balance equations (Equations 19–21) can be written as follows:

$$D_{BH_{2}^{2+}}\frac{d^{2}\left[BH_{2}^{2+}\right]}{d^{2}x}+D_{{\text{BH}}^{+}}\frac{d^{2}\left[{\text{BH}}^{+}\right]}{d^{2}x}+D_{B}\frac{d^{2}\left[B\right]}{d^{2}x}=0,$$ (19)

$$D_{HA}\frac{d^{2}\left[HA\right]}{d^{2}x}+D_{A^{-}}\frac{d^{2}\left[A^{-}\right]}{d^{2}x}=0,$$ (20)

$$D_{BH_{2}^{2+}}\frac{d^{2}\left[BH_{2}^{2+}\right]}{d^{2}x}+D_{H^{+}}\frac{d^{2}\left[H^{+}\right]}{d^{2}x}+D_{HA}\frac{d^{2}\left[HA\right]}{d^{2}x}-D_{{\text{OH}}^{-}}\frac{d^{2}\left[{\text{OH}}^{-}\right]}{d^{2}x}-D_{B}\frac{d^{2}\left[B\right]}{d^{2}x}-D_{A^{-}}\frac{d^{2}\left[A^{-}\right]}{d^{2}x}=0.$$ (21)

Integrating above equations yields Equations 22–24:

$$D_{BH_{2}^{2+}}\frac{d\left[BH_{2}^{2+}\right]}{\text{dx}}+D_{{\text{BH}}^{+}}\frac{d\left[{\text{BH}}^{+}\right]}{\text{dx}}+D_{B}\frac{d\left[B\right]}{\text{dx}}=C_{1},$$ (22)

$$D_{HA}\frac{d\left[HA\right]}{\text{dx}}+D_{A^{-}}\frac{d\left[A^{-}\right]}{\text{dx}}=C_{2},$$ (23)

$$D_{BH_{2}^{2+}}\frac{d\left[BH_{2}^{2+}\right]}{dx}+D_{H^{+}}\frac{d\left[H^{+}\right]}{dx}+D_{HA}\frac{d\left[HA\right]}{\text{dx}}-D_{{\text{OH}}^{-}}\frac{d\left[{\text{OH}}^{-}\right]}{dx}-D_{B}\frac{d\left[B\right]}{dx}-D_{A^{-}}\frac{d\left[A^{-}\right]}{\text{dx}}=C_{3}.$$ (24)

According to Fick’s first law of diffusion, the $D_{i}\frac{dC_{i}}{\text{dt}}$ terms in the equations above represent the fluxes (J) of the respective species. By charge neutrality, the sum of the fluxes of species reacting as acids must equal the sum of the fluxes of species reacting as bases, as shown in Equation 25:

$$\sum \left(J_{BH_{2}^{2+}}+J_{{\text{BH}}^{+}}+J_{H^{+}}\right)=\sum \left(J_{{\text{OH}}^{-}}+J_{A^{-}}\right).$$ (25)

Integrating Equations 22–24 with respect to x yields Equations 26–28:

$$D_{BH_{2}^{2+}}\left[BH_{2}^{2+}\right]+D_{{\text{BH}}^{+}}\left[{\text{BH}}^{+}\right]+D_{B}\left[B\right]=C_{1}x+C_{4},$$ (26)

$$D_{HA}\left[HA\right]+D_{A^{-}}\left[A^{-}\right]=C_{2}x+C_{5},$$ (27)

$$D_{BH_{2}^{2+}}\left[BH_{2}^{2+}\right]+D_{H^{+}}\left[H^{+}\right]+D_{\text{HA}}\left[HA\right]-D_{{\text{OH}}^{-}}\left[{\text{OH}}^{-}\right]-D_{B}\left[B\right]-D_{A^{-}}\left[A^{-}\right]=C_{3}x+C_{6}.$$ (28)

The boundary conditions for the differential equations are defined as follows:

At x = 0 (at the surface of the dissolving solid),

$$\left[B\right]={\left[B\right]}_{0}\left(\text{intrinsicsolubilityofthedrug}\right),$$

$$\left[{\text{BH}}^{+}\right]={\left[{\text{BH}}^{+}\right]}_{0}\text{unknown},$$

$$\left[BH_{2}^{2+}\right]={\left[BH_{2}^{2+}\right]}_{0}\text{unknown},$$

$$\left[A^{-}\right]={\left[A^{-}\right]}_{0}\text{unknown},$$

$$\left[H^{+}\right]={\left[H^{+}\right]}_{0}\text{unknown},$$

$$\left[{\text{OH}}^{-}\right]={\left[{\text{OH}}^{-}\right]}_{0}\text{unknown},$$

where the subscript 0 represents the surface concentration of the respective species.

At x = h (the boundary between the diffusion layer and the bulk medium),

$$\left[B\right]={\left[B\right]}_{h}=0\left(\text{sinkcondition}\right),$$

$$\left[{\text{BH}}^{+}\right]={\left[{\text{BH}}^{+}\right]}_{h}=0\left(\text{sinkcondition}\right),$$

$$\left[BH_{2}^{2+}\right]={\left[BH_{2}^{2+}\right]}_{h}=0\left(\text{sinkcondition}\right),$$

$$\left[A^{-}\right]={\left[A^{-}\right]}_{h}=0\left(\text{sinkcondition}\right),$$

$$\left[H^{+}\right]={\left[H^{+}\right]}_{h}\text{known},$$

$$\left[{\text{OH}}^{-}\right]={\left[{\text{OH}}^{-}\right]}_{h}\text{known},$$

where the subscript h represents the bulk concentration of the respective species.

Applying the above boundary conditions to Equations 26–28, at x = 0, Equations 29–31 can be obtained:

$$D_{BH_{2}^{2+}}{\left[BH_{2}^{2+}\right]}_{0}+D_{{\text{BH}}^{+}}{\left[BH^{+}\right]}_{0}+D_{B}{\left[B\right]}_{0}=C_{4},$$ (29)

$$D_{\text{HA}}{\left[HA\right]}_{0}+D_{A^{-}}{\left[A^{-}\right]}_{0}=C_{5},$$ (30)

$$D_{BH_{2}^{2+}}{\left[BH_{2}^{2+}\right]}_{0}+D_{H^{+}}{\left[H^{+}\right]}_{0}+D_{\text{HA}}{\left[HA\right]}_{0}-D_{{\text{OH}}^{-}}{\left[OH^{-}\right]}_{0}-D_{B}{\left[B\right]}_{0}-D_{A^{-}}{\left[A^{-}\right]}_{0}=C_{6}.$$ (31)

Applying the above boundary conditions to Equations 26–28, at x = h, we obtain Equations 32–34 :

$$0=C_{1}h+C_{4},$$ (32)

$$0=C_{2}h+C_{5},$$ (33)

$$D_{H^{+}}{\left[H^{+}\right]}_{h}-D_{{\text{OH}}^{-}}{\left[OH^{-}\right]}_{h}=C_{3}h+C_{6}.$$ (34)

Subtracting Equations 29–32 results in Equation 35:

$$D_{BH_{2}^{2+}}{\left[BH_{2}^{2+}\right]}_{0}+D_{{\text{BH}}^{+}}{\left[BH^{+}\right]}_{0}+D_{B}{\left[B\right]}_{0}=-C_{1}h.$$ (35)

Subtracting Equations 30–33 results in Equation 36:

$$D_{HA}{\left[HA\right]}_{0}+D_{A^{-}}{\left[A^{-}\right]}_{0}=-C_{2}h.$$ (36)

Subtracting Equations 31–34 results in Equation 37:

$$D_{BH_{2}^{2+}}{\left[BH_{2}^{2+}\right]}_{0}+D_{\text{HA}}{\left[\text{HA}\right]}_{0}+D_{H^{+}}{\left[H^{+}\right]}_{0}-{\left[H^{+}\right]}_{h}\backslash )-D_{{\text{OH}}^{-}}\left({\left[OH^{-}\right]}_{0}-{\left[OH^{-}\right]}_{h}\right)-D_{B}{\left[B\right]}_{0}-D_{A^{-}}{\left[A^{-}\right]}_{0}=-C_{3}h.$$ (37)

Combining Equations 35– 3735– 37^35– 37^ and successive substitution to solve the equations for a single unknown term ( ${\left[H^{+}\right]}_{0}$ ), yields the Equation 38:

$$D_{A}\left(\frac{D_{HA}{\left[HA\right]}_{h}+D_{A}{\left[A\right]}_{h}}{D_{A}+D_{HA}\left(\frac{{\left[H^{+}\right]}_{0}}{K_{a3}}\right)}-{\left[A\right]}_{h}\right)+D_{OH}\left(\frac{K_{w}}{{\left[H^{+}\right]}_{0}}-{\left[OH\right]}_{h}\right)=D_{BH}\left(\frac{{\left[B\right]}_{0}{\left[H^{+}\right]}_{0}}{K_{a1}}-{\left[BH^{+}\right]}_{h}\right)+2D_{BH_{2}^{2+}}\left(\frac{{\left[B\right]}_{0}{{\left[H^{+}\right]}_{0}}^{2}}{K_{a1}K_{a2}}-{\left[BH_{2}^{2+}\right]}_{h}\right)+D_{H}\left({\left[H^{+}\right]}_{0}-{\left[H^{+}\right]}_{h}\right).$$ (38)

After cross-multiplication with ${\left[H^{+}\right]}_{0}$ and rearranging above equation yields the final format of fourth power polynomial equation (Equation 39) as a function of the concentration of the hydrogen ion at the solid surface:

$$p{{\left[H^{+}\right]}_{0}}^{4}+q{{\left[H^{+}\right]}_{0}}^{3}+r{{\left[H^{+}\right]}_{0}}^{2}+s{\left[H^{+}\right]}_{0}+t=0,$$ (39)

where

$$p=2D_{BH_{2}^{2+}}D_{HA}{\left[B\right]}_{0},$$

$$q=D_{HA}D_{H}K_{a1}K_{a2}+D_{HA}D_{BH^{+}}{\left[B\right]}_{0}K_{a2}+2D_{A}D_{BH_{2}^{2+}}{\left[B\right]}_{0}K_{a3},$$

$$r=-{2D}_{HA}D_{{BH}_{2}^{2+}}{\left[{BH}_{2}^{2+}\right]}_{h}K_{a1}K_{a2}+D_{A}D_{H}K_{a1}K_{a2}K_{a3}+D_{HA}D_{OH-}\;{\left[OH\right]}_{h}K_{a1}K_{a2}+D_{A}D_{BH^{+}}{\left[B\right]}_{0}K_{a2}K_{a3}+D_{HA}D_{A}{\left[A\right]}_{h}K_{a1}K_{a2}-D_{HA}D_{H}{\left[H^{+}\right]}_{h}K_{a1}K_{a2}-D_{HA}D_{BH^{+}}{\left[BH^{+}\right]}_{h}K_{a1}K_{a2}$$

$$s=-{2D}_{A}D_{{BH}_{2}^{2+}}{\left[{BH}_{2}^{2+}\right]}_{h}K_{a1}K_{a2}K_{a3}+D_{A}D_{OH-}{\left[OH\right]}_{h}K_{a1}K_{a2}K_{a3}-D_{A}D_{H}{\left[H^{+}\right]}_{h}K_{a1}K_{a2}K_{a3}-D_{A}D_{BH^{+}}{\left[BH^{+}\right]}_{h}{\left[B\right]}_{0}K_{a1}K_{a2}K_{a3}-D_{HA}D_{A}{\left[HA\right]}_{h}K_{a1}K_{a2}K_{a3}-D_{HA}D_{OH^{-}}K_{w}K_{a1}K_{a2}$$

$$t=-D_{A}D_{OH^{-}}K_{w}K_{a1}K_{a2}K_{a3}.$$

The polynomial equation was numerically solved using the “roots” function of MATLAB software.

The detailed derivation of the mass transport equations for other scenarios, i.e., a diprotic weak base with a monoprotic acidulant under buffered conditions and a diprotic weak base with a diprotic acidulant under unbuffered and buffered conditions, is provided in appendices of the supplementary document (Supplementary Sections S1, S2).

### 2.1 Application of the model to study the dissolution kinetics of a drug product containing a pH-modifying excipient

The expanded model with pH-modifying excipients was used to study the impact of surface pH on the dissolution behavior of immediate release (IR) solid oral formulations containing a weakly basic model drug, entrectinib, in combination with acidifying excipients (monoprotic and diprotic acid). Entrectinib is a lipophilic base, classified as a Biopharmaceutical Classification System (BCS) class II drug based on its low aqueous solubility and good permeability. It is highly soluble at low pH. However, its solubility significantly decreases as pH increases, posing a risk of pH-dependent drug–drug interactions when administered with gastric-acid-reducing agents. To address this risk, entrectinib capsules were developed with distinct acidifying excipients: formulation F1 containing a monoprotic acidifier, F2 with a diprotic acidifier, and F0 without any acidifier (Parrott et al., 2020).

Table 1 outlines USP 2 dissolution testing conditions for the three different formulations of entrectinib 200 mg capsules. These conditions were established during dissolution method development to aid the drug development program. Although the original analysis by Mooney et al. was developed for the dissolution of a compressed powder exposed to external media via a rotating disc, our analysis applies these fundamental interfacial principles to multiparticulate dissolution from a capsule. Once the capsule shell ruptures and disintegrates, the local microenvironment at the solid–liquid interface of the discrete primary particles dictates the dissolution rate. To place this within the larger frame of *in vitro* dissolution testing, our mathematical model was used to calculate the precise surface pH. This surface pH was then integrated into commercial software platforms used for *in vitro* dissolution modeling: SIVA V5 [Simcyp *in vitro* (data) Analysis Toolkit Version 5.0] from Certara Simcyp and DDDPlus™ V5 (Dose Disintegration and Dissolution Plus, Version 5) from Simulations Plus Inc. SIVA and DDDPlus are designed for *in vitro* data modeling.

**TABLE 1:** Studied formulations and dissolution conditions.

Formulation code	Formulation characteristics	Dissolution condition/Medium
F0	Without an acidifier Dose: 200 mg Drug substance particle size distribution: D10 = 8 μm, D50 = 30 μm, and D90 = 121 µm	750 mL of 0.1 M HCl pH 1 paddle 75 rpm
		500 mL of 0.0001 M HCl pH 4 paddle 75 rpm
F1	With a monoprotic acidifier Dose: 200 mg Acidifier: Betaine HCl Molar ratio (drug: acidifier): 1:1.5 Drug substance particle size distribution: D10 = 4 μm, D50 = 19 μm, and D90 = 49 µm	750 mL of 0.1 M HCl pH 1 paddle 75 rpm
		500 mL of 0.0001 M HCl pH 4 paddle 75 rpm
		500 mL of SGFsp pH 1.2 paddle 50 rpm
		500 mL of acetate buffer pH 4.5 paddle 50 rpm
F2	With a diprotic acidifier Dose: 200 mg Acidifier: Tartaric acid Molar ratio (drug: acidifier): 1:1 Drug substance particle size distribution: D10 = 4 μm, D50 = 25 μm, and D90 = 65 µm	750 mL of 0.1 M HCl pH 1 paddle 75 rpm
		500 mL of 0.0001 M HCl pH 4 paddle 75 rpm
		500 mL of SGFsp pH 1.2 paddle 50 rpm
		500 mL of acetate buffer pH 4.5 paddle 50 rpm

The SIVA toolkit models dissolution using the diffusion layer model (DLM), originally developed by Wang and Flanagan (2002) to describe drug release from monodisperse spherical particles under both sink and non-sink conditions. The model incorporates key parameters, including particle radius, particle number, effective diffusion coefficient, diffusion layer thickness, surface solubility, and bulk drug concentration, to capture dissolution dynamics (Pathak et al., 2017; Klumpp and Dressman, 2020).

DDDPlus™ predicts dissolution behavior by numerically solving nonlinear equations derived from equilibrium constants, mass balance, and ionic balance relationships that capture the relevant ionic equilibria in the chosen medium (Parrott et al., 2020; Almukainzi et al., 2015). The software provides multiple dissolution model options, all grounded in the classical diffusion layer theory. By default, the mass transfer model is used, which assumes forced convection around spherical particles and calculates a mass transfer coefficient based on fluid velocity and medium viscosity. Alternative models include the Nernst–Brunner, Johnson–spherical, Johnson–cylindrical, and intrinsic dissolution models. The intrinsic dissolution model, in particular, is used to estimate dissolution at the surface of a non-disintegrating rotating disk.

By utilizing these software packages, we account for macroscopic system factors such as fluid hydrodynamics driven by paddle speed, media volume, dose, and particle size distribution, while our external mathematical model supplies the critical surface pH parameter.

#### 2.1 Dissolution model validation performance

To evaluate the dissolution model performance under bulk pH and surface pH hypothesis, the average fold error (AFE) and absolute average fold error (AAFE) were calculated based on the percent of drug dissolved at the measured time points for each case (Parrott et al., 2022). The AFE reveals whether the predictions tend to underestimate or overestimate the observed values. The AAFE provides a measure of the absolute prediction error to observed data.

## 3 Results

### 3.1 Surface pH prediction in the presence of unbuffered media

The surface pH of entrectinib free base formulation without an acidifying agent (F0) was estimated based on its physicochemical properties (Supplementary Table S1) using the derived polynomial equation, provided in Supplementary Sections S1, S2. Model predictions are shown in Figure 2. In unbuffered acidic media (pH 1–5), acid–base reactions lead to an elevated surface pH compared to the bulk pH, which is attributed due to the inherent self-buffering property of entrectinib. However, in unbuffered neutral and basic media, the free base does not react, resulting in a surface pH, which is equal to the bulk pH, as expected for a weak base compound. Moreover, the addition of a pH-modifying excipient, such as a monoprotic or diprotic acidulant (F1 and F2, respectively), significantly overcomes entrectinib’s self-buffering capability.

**FIGURE 2:**
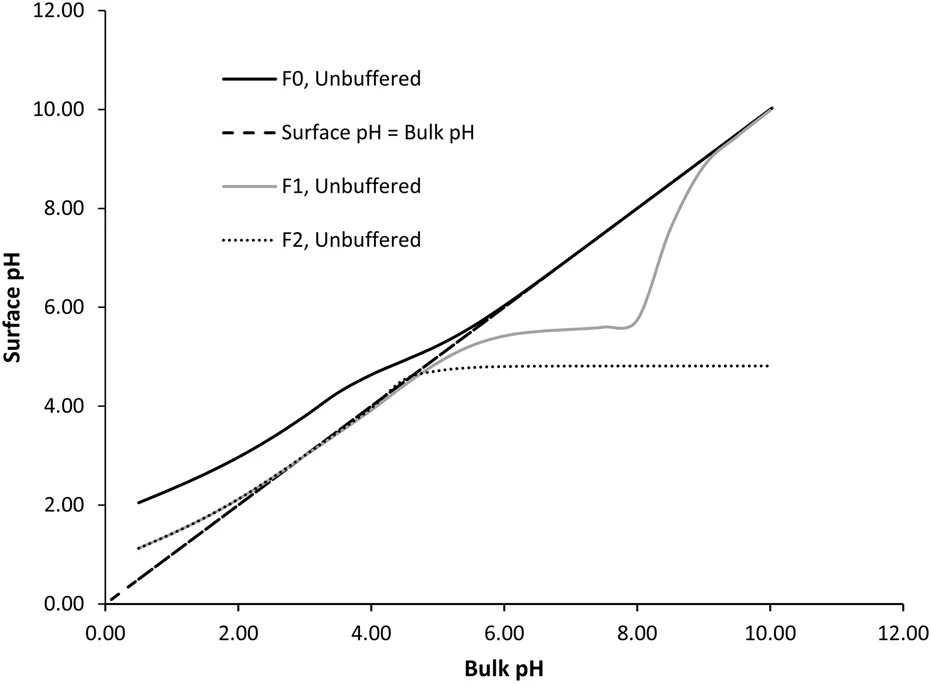
Surface pH vs. bulk pH of entrectinib formulations in an unbuffered aqueous medium.

### 3.2 Surface pH prediction in the presence of buffered media

Using the derived equation (see Supplementary Sections S1, S2) and based on the physicochemical properties (Supplementary Table S1), the surface pH of entrectinib formulations (F0, F1, and F2) was predicted under acetate buffered conditions across various buffer strengths. Figure 3 illustrates the impact of acetate buffer species on the surface pH of entrectinib formulations as a function of buffer concentration. The surface pH varies depending on both the acidifying excipients and buffering agent concentrations. In the absence of the acidifier (F0), the surface pH is only slightly higher (4.59) than the bulk pH of 4.5 as the buffer moderates entrectinib’s self-buffering effect. In the monoprotic acidifier formulation (F1), the surface pH is slightly lower (4.43) than the bulk pH of 4.5, due to the presence of a weak acid in acid–base reactions within the diffusion layer. In the diprotic acidifier formulation (F2), surface pH significantly decreases (3.95–2.93) compared to the bulk pH of 4.5, due to reactions between the strong acidulant and the buffer’s basic components at the dissolving surface (Figure 3). This reaction increases the [H+] ion concentration in the diffusion layer, lowering the surface pH. Due to its lower basicity, the acetate buffer cannot effectively counteract this acidification, particularly in the diprotic acidifier case.

**FIGURE 3:**
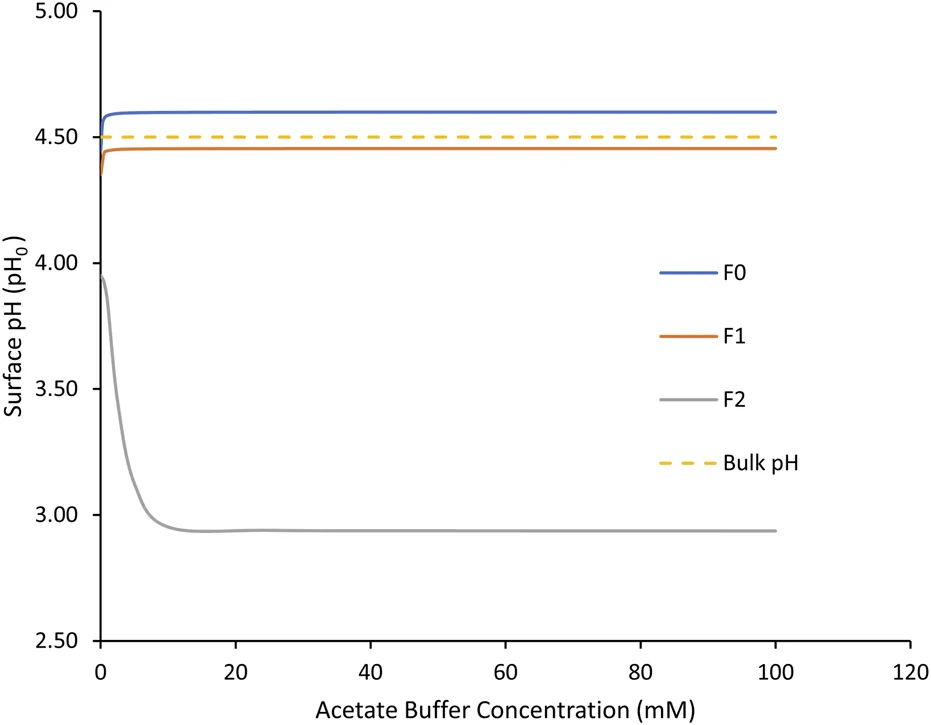
Theoretical predictions of surface pH for entrectinib formulations as a function of acetate pH 4.5 buffer concentration.

### 3.3 Dissolution modeling of entrectinib’s F0 formulation (without an acidifying agent)

Both SIVA and DDDPlus estimate dissolution based on the diffusion layer model equations in the specified dissolution media. The *in vitro* dissolution studies displayed complete dissolution within 15 min in an unbuffered medium at pH 1, which was accurately replicated using both software platforms. At the lowest pH, despite the greater difference between bulk and surface pH, both predicted and observed dissolution rates remained very rapid, resulting in minimal prediction errors. As pH increases, solubility decreases, and larger variations in dissolution can be observed depending on whether bulk or surface pH is considered.

However, using bulk pH as an input to the model in an unbuffered medium with an initial pH of 4 significantly overestimated the rate and extent of dissolution (Figure 4). Measurement of the medium pH after dissolution of F0 (formulation without an acidifying agent) showed an increase in bulk pH from 4 to 5.2. This increase is due to the self-buffering effect of entrectinib. Using surface pH enables more accurate predictions of the observed data, with all performance indicators calculated for these predictions (Table 2).

**FIGURE 4:**
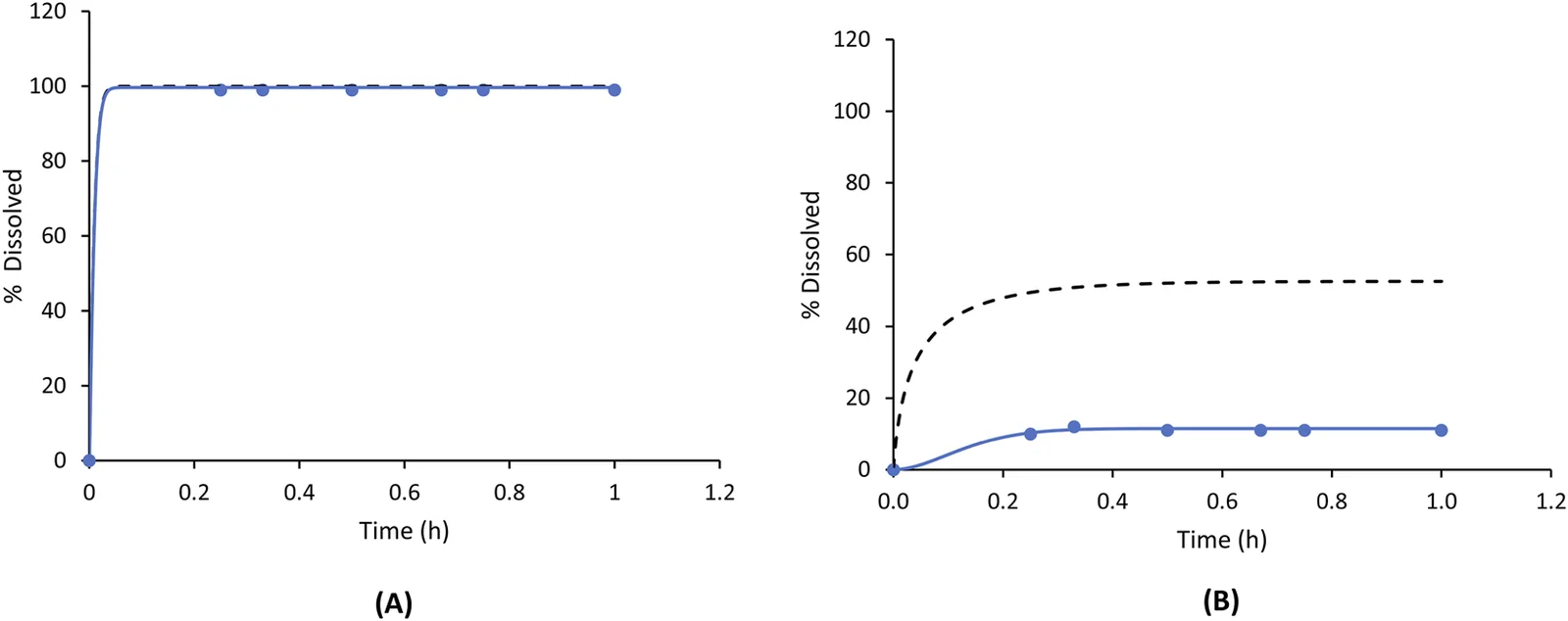
Predicted dissolution profiles using the SIVA toolkit for F0 formulation (without an acidifying agent) with **(A)** 0.1 M HCl pH 1 media and **(B)** 0.0001 M HCl pH 4 unbuffered media. The dashed black line represents the model prediction using bulk pH, while the solid blue line corresponds to the prediction using surface pH, and filled blue circles denote the observed data.

**TABLE 2:** AFE and AAFE for the prediction of percent dissolved using the SIVA toolkit.

Formulation code	Dissolution condition	AFE bulk pH	AAFE bulk pH	AFE surface pH	AAFE surface pH
F0	750 mL of 0.1 M HCl pH 1 paddle 75 rpm	1.01	1.01	1.01	1.01
	500 mL of 0.0001 M HCl pH 4 paddle 75 rpm	4.70	4.70	1.02	1.05
F1	750 mL of 0.1 M HCl pH 1 paddle 75 rpm	1.00	1.00	1.00	1.00
	500 mL of 0.0001 M HCl pH 4 paddle 75 rpm	0.55	1.81	1.02	1.02
	500 mL of SGFsp pH 1.2 paddle 50 rpm	1.00	1.01	1.00	1.01
	500 mL of acetate buffer pH 4.5 paddle 50 rpm	0.95	1.09	1.03	1.04
F2	750 mL of 0.1 M HCl pH 1 paddle 75 rpm	0.98	1.14	0.98	1.14
	500 mL of 0.0001 M HCl pH 4 paddle 75 rpm	0.48	2.10	0.73	1.42
	500 mL of SGFsp pH 1.2 paddle 50 rpm	0.79	1.27	0.79	1.27
	500 mL of acetate buffer pH 4.5 paddle 50 rpm	0.55	1.80	0.72	1.39

### 3.4 Dissolution modeling of entrectinib formulation F1 (with a monoprotic acidifying agent)

*In vitro* dissolution studies showed complete dissolution of the F1 formulation within 15 min in both, an unbuffered medium at pH 1 and SGFsp at pH 1.2, which was accurately captured using the two software platforms. As previously discussed, at this low pH, despite a difference between the bulk pH and the surface pH, the predicted and observed dissolution rates were both very rapid, leading to minimal prediction errors regardless of which pH value was applied.

As the pH increased, solubility decreased, and the impact of bulk versus surface pH on the dissolution rate became more pronounced. When the bulk pH alone was used in the model with an initial unbuffered pH 4 and in a 50 mM acetate buffer pH 4.5, the rate and extent of dissolution were significantly underestimated (Figure 5). This discrepancy arises due to the model not accounting for the monoprotic acidifier’s effect, which stabilizes an acidic environment at the solid–liquid interface and enhances drug ionization. Inputting the estimated surface pH into the model accurately predicted the dissolution behavior.

**FIGURE 5:**
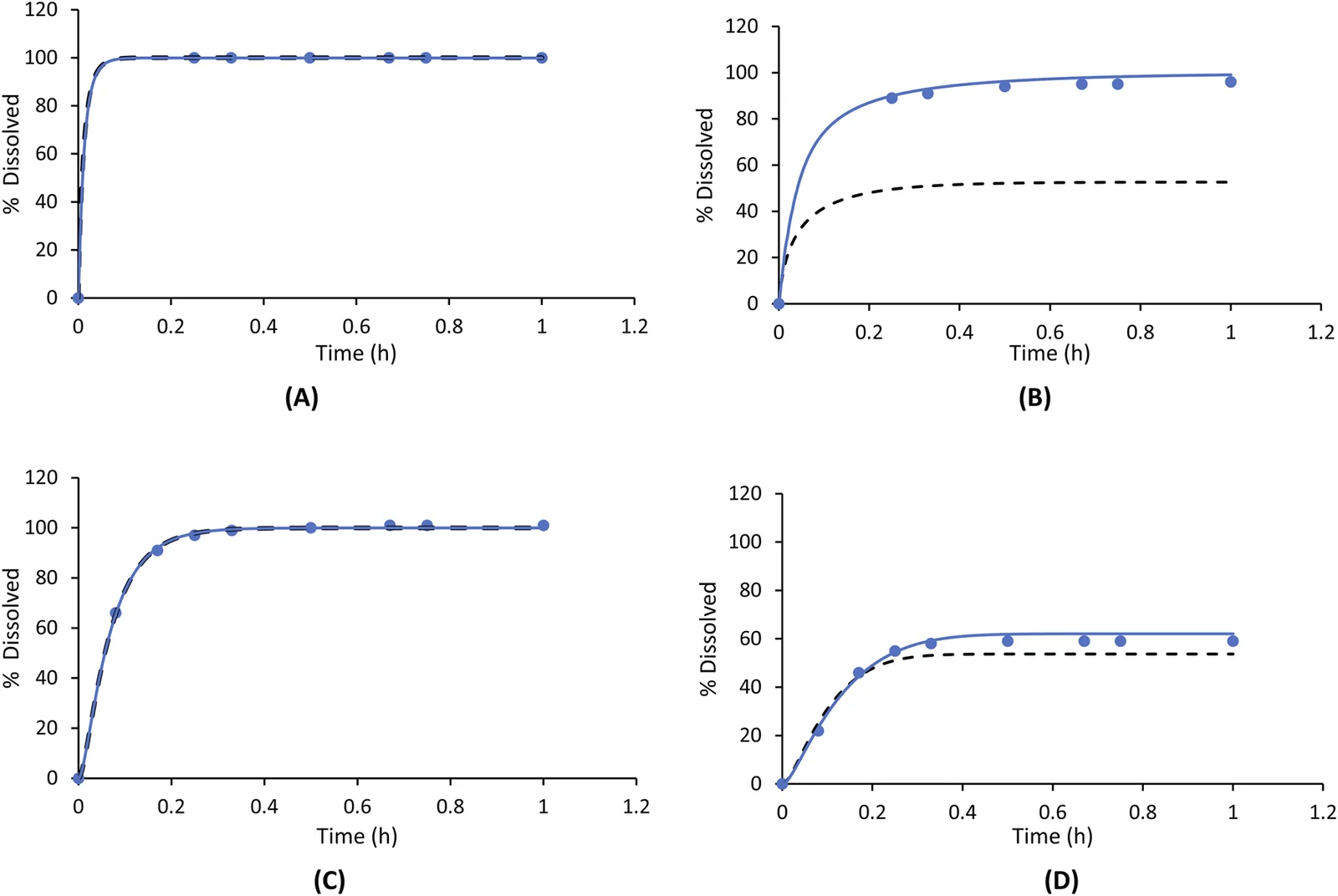
Predicted dissolution profiles using the SIVA toolkit for F1 formulation (with a monoprotic acidifying agent) were evaluated in unbuffered media: **(A)** 0.1 M HCl, pH 1; **(B)** 0.0001 M HCl, pH 4; **(C)** SGF pH 1.2; and **(D)** in 50 mM acetate buffer, pH 4.5. The dashed black line represents the model prediction using bulk pH, while the solid blue line corresponds to the prediction using surface pH, and filled blue circles denote the observed data.

### 3.5 Dissolution modeling of entrectinib’s F2 formulation (with a diprotic acidifying agent)

In the F2 formulation, differences in the capsule material led to a slower disintegration (disintegration time of 14 min was estimated). As discussed, at the low pH, although bulk and surface pH differed, the observed and predicted dissolution rates were very rapid, leading to minimal prediction errors regardless of which pH value was used.

As the pH increased, solubility decreased, making the influence of bulk versus surface pH on dissolution rates more noticeable. Using only bulk pH in the model with an initial unbuffered pH of 4 and in a 50 mM acetate buffer at pH 4.5 led to significant underestimation of dissolution rates and extent (Figure 6). This mismatch arose because the model did not consider the diprotic acidifier’s role in maintaining an acidic environment at the solid–liquid interface, which promotes drug ionization. Incorporating the estimated surface pH into the model provided an accurate prediction of the dissolution behavior. Similar results were obtained using both software platforms; the data represented here were generated using the SIVA V5 toolkit, while the results from DDD Plus are provided in the Supplementary Material (Supplementary Figures S1, S2, S3).

**FIGURE 6:**
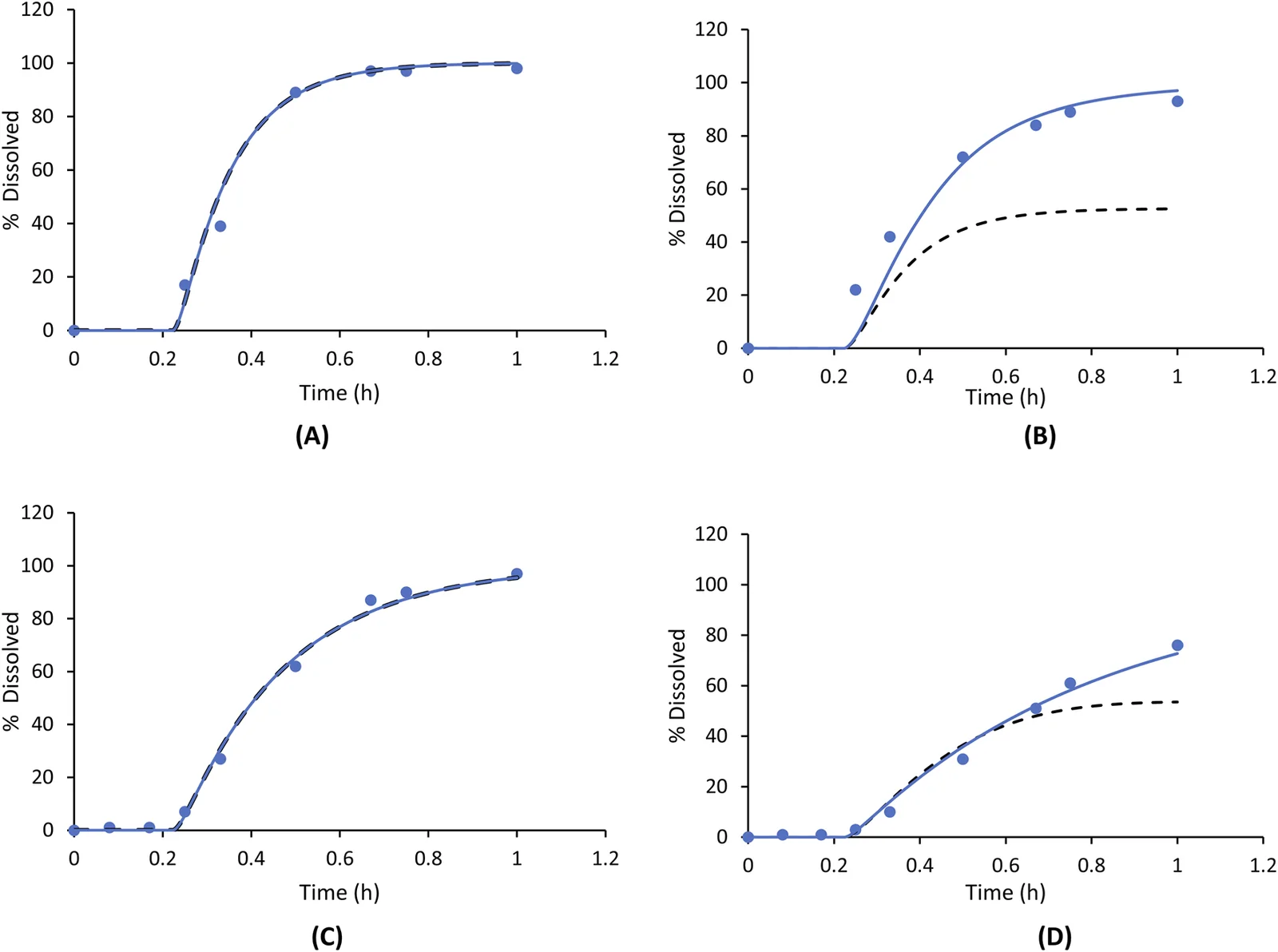
Predicted dissolution profiles using the SIVA toolkit for F2 formulation (with a diprotic acidifying agent) in unbuffered media: **(A)** 0.1 M HCl, pH 1; **(B)** 0.0001 M HCl, pH 4; **(C)** SGF pH 1.2; and **(D)** 50 mM acetate buffer, pH 4.5. The dashed black line represents the model prediction using bulk pH, while the solid blue line corresponds to the prediction using surface pH, and filled blue circles denote the observed data.

### 3.6 Effect of the dissolved drug on surface solubility and the dissolution rate

The solubility and dissolution rate of acidic or basic drugs can be significantly influenced by changes in the surface pH. This study focuses on entrectinib, a BCS Class II weak base, as a case example to explore these effects. Entrectinib free base capsules without an acidifying agent demonstrate a decrease in AUC by 25% and Cmax by 23% when co-administered with a proton pump inhibitor (PPI) (Parrott et al., 2020). This reduction is attributed to decreased solubility and dissolution in the gastric environment at elevated pH levels. However, incorporating a strong acidifying agent into the formulation (e.g., F06) can effectively mitigate this issue (Parrott et al., 2020).

Surface pH can be estimated by measuring the pH of a saturated drug solution in a relevant medium. Although this is feasible for entrectinib free base, formulations containing acidifying agents present challenges for direct surface pH measurement due to their complexity. Therefore, we developed a customized surface pH calculator [based on the studies of Mooney et al. (1981a) and Mooney et al. (1981b)] to predict the surface pH of entrectinib with an acidifier as a pH-modifying excipient. Modeling dissolution rates using bulk pH rather than surface pH may lead to discrepancies, particularly when there is a significant difference between the two. To address this, surface solubility can be integrated into oral absorption modeling tools by either setting bulk pH equivalent to surface pH or directly inputting surface solubility as a function of pH into software such as Simcyp.

Surface pH changes are particularly impactful on oral absorption predictions when dissolution is the rate-limiting step. For entrectinib, sensitivity analyses revealed that surface pH modulation depends on the drug’s solid form and the type of acidifying agent included in the formulation (e.g., monoprotic vs. diprotic acids). The extent to which these surface pH changes translate into *in vivo* and *in silico* outcomes, such as alterations in AUC and Cmax, depends on the interaction between the drug, formulation, and gastrointestinal fluid properties.

Ultimately, oral drug performance, driven by solubility and its impact on dissolution, precipitation, and permeability, is highly influenced by the interplay between the drug, its formulation, and the GI environment. These factors can be accurately predicted using specialized *in vitro* tools combined with physiologically based biopharmaceutics modeling (PBBM) (Pepin et al., 2024; Pepin et al., 2021).

## 4 Discussion

The oral absorption of drugs from the GI tract is a complex process influenced by several factors, including, but not limited to, the drug’s physicochemical properties, formulation characteristics, and the underlying physiological properties of the GI tract (Shah et al., 2014; Mudie et al., 2010). The drug dissolution rate is influenced by physicochemical properties such as pKa, particle size, and intrinsic solubility. Physiological conditions such as gastrointestinal motility, hydrodynamic conditions in the GI tract, buffer species, buffer strength, bile salt concentrations, and luminal pH also play a critical role (Mitra and Kesisoglou, 2013). Additionally, excipients in the formulation can influence the microenvironmental pH at the particle surface, thereby affecting dissolution. Weakly basic drugs, in particular, show pH-dependent solubility: they dissolve readily in the acidic gastric environment but may precipitate as they enter the more neutral pH of the intestine. Thus, the pH transition from the acidic stomach to the small intestine can greatly impact the solubility of a weakly basic drug (Pepin et al., 2024). pH-modifying excipients, such as acidifying or basifying agents, significantly modify the microenvironmental pH around the drug particles. By enhancing solubility and dissolution rates, these excipients can significantly improve the oral bioavailability of drugs. Building on this concept, mechanistic models have been developed to quantitatively capture how such pH modifications within the diffusion layer influence drug dissolution and ionization dynamics.

The original model by Mooney et al. effectively predicts dissolution rates and surface pH profiles by modeling diffusion-controlled mass transport and instantaneous ionization equilibria (e.g., HA ⇌ H^+^ + A^−^) within the diffusion layer, validated for compounds such as benzoic acid under unbuffered and buffered conditions (Mooney et al., 1981a; Mooney et al., 1981b). However, its inability to account for pH-modifying excipients restricts its applicability to drug products in which excipients are strategically used to tailor the microenvironment pH, thereby enhancing solubility and bioavailability.

In the present study, the original framework of the Mooney model was extended, by including the diffusion and reaction of excipient species within the diffusion layer. The inclusion of excipient dynamics enhances the model predictive accuracy for complex formulations, particularly those designed for poorly soluble drugs such as entrectinib.

The self-buffering effect noted with the F0 formulation (no acidifying agent) offers particularly important insights into the inherent behavior of weak base drugs during their dissolution. In other formulations, the effectiveness of the acidifier in maintaining lower surface pH is critical given the steep solubility–pH relationship. The mechanistic insights analyzed in this study highlight several critical areas, in which existing dissolution modeling techniques need significant enhancement especially in the case of formulations with pH-modifying excipients. The differences observed between bulk and surface pH predictions, especially in the F1 and F2 formulations with monoprotic and diprotic acidifying agents, respectively, highlight the limitations of present models that overlook the contribution of the acidifier species in dynamic chemical equilibria formed within the diffusion layer. Furthermore, a key finding of this investigation is that surface pH is the key driver of solubility and dissolution kinetics for weak-base drugs. Therefore, when using bulk pH and overlooking the impact of acidifying agents on the pH in the diffusion layer, the predictions differed from experimental dissolution results, with average fold errors between 0.48 and 4.70 and average absolute fold errors ranging from 1.80 to 4.70 across various pH levels. In contrast, the inclusion of surface pH calculations in the presence of the pH-modifying species significantly boosted model performance, reaching average fold errors of 0.72–1.03 and average absolute fold errors between 1.00 and 1.42, indicating a notable improvement in predictive precision. Both software platforms, SIVA and DDDPlus, produced comparable results in modeling the *in vitro* dissolution profiles. Although their interfaces differ, both initially failed to account for the influence of surface pH changes caused by pH-modifying excipients, resulting in similar predictions. However, upon incorporating the surface pH estimated using the extended Mooney model into SIVA and DDDPlus, both platforms accurately captured the dissolution behavior under the tested conditions.

The validation of the extended model against experimental dissolution data demonstrated its predictive capability compared to approaches that rely solely on bulk pH. The model successfully captured the enhanced dissolution rates observed for entrectinib in the presence of acidifying agents, accurately predicting the magnitude of surface pH reduction and its impact on drug solubility at the solid–liquid interface. This validation confirmed that the mathematical extensions preserved the mechanistic foundation of the original Mooney model while expanding its applicability to clinically relevant formulation scenarios.

Overall, the extension of the Mooney model represents a significant step toward more comprehensive mechanistic modeling of pharmaceutical dissolution processes. By incorporating the chemical complexity of modern drug formulations while maintaining the rigorous theoretical foundation of the original framework, the extended model bridges the gap between fundamental dissolution science and practical formulation development. This advancement enables more accurate prediction of formulation effects on drug dissolution and supports the development of robust formulations that maintain consistent performance across diverse physiological conditions. The ability to predict surface pH based on fundamental physicochemical parameters such as drug pKa, intrinsic solubility, and diffusion coefficients offers pharmaceutical scientists a powerful tool for rational formulation design. This mechanistic approach enables the optimization of excipient type and amount to achieve target dissolution profiles without extensive empirical screening, potentially accelerating formulation development timelines and reducing development costs.

Additionally, the investigation exposes significant limitations in commercially available dissolution modeling and physiologically based pharmacokinetic (PBPK) modeling software, which generally fail to include surface pH calculations for weak-base drugs that are formulated with pH-modifying excipients. This deficiency in modeling abilities poses a considerable challenge to the adoption of model-informed drug development strategies for complex formulations. Incorporating surface pH factors into dissolution and PBPK models would allow for better forecasting of formulation impacts on drug uptake and could assist in regulatory filings for formulation modifications or bioequivalence exemptions.

A limitation of the present model is that it describes ionic reactions in terms of concentrations rather than thermodynamic activities. In our experimental dissolution studies, the ionic strengths reached up to approximately 0.1 M (e.g., in the 0.1 M HCl medium and in the 50 mM acetate buffer following the dissolution of the drug and acidulant). At these ionic strengths, mean ionic activity coefficients deviate from unity. If a thermodynamically adjusted equilibrium solution utilizing the extended Debye–Huckel approximation were implemented, the effective activities of the ionic species, particularly multivalent ions, would be lowered. This would result in slightly shifted apparent pKa values for both the drug and the acidulant at the solid–liquid interface. Consequently, incorporating thermodynamic activity corrections would likely yield a minor but meaningful refinement in the predicted surface pH and subsequently solubility estimations, potentially further minimizing residual prediction errors at higher ionic strengths.

In summary, this research offers strong proof of the crucial role of surface pH influences in the dissolution of weak-base drugs and shows the insufficiency of conventional models that overlook the impact of the surface pH-modifying agents. The mechanistic insights uncovered by this study have substantial implications for formulation development, regulatory evaluation, and our core understanding of drug dissolution processes. The improved model created in this study could serve as a useful resource for pharmaceutical researchers and may aid in the creation of more efficient and dependable drug delivery systems. Nevertheless, ongoing research is essential to overcome the limitations recognized in this study and to expand these results to a wider variety of pharmaceutical systems and applications.

## 5 Conclusion

In this study, we introduce an advanced mass transport model that incorporates surface pH effects to predict the dissolution of weakly basic drugs in formulations containing acidifying excipients. An equilibrium-based mass transport model was developed to predict the dissolution of weakly basic drugs with added pH-modifying excipients, such as monoprotic and diprotic acidifiers. The model is highly effective in highlighting the importance of surface pH—an aspect often overlooked in traditional dissolution and pharmacokinetic modeling. This model serves as a tool for understanding how acidifying agents influence the oral delivery of immediate-release drug products containing weak bases. Across all conditions and formulation variants tested, relying on bulk pH alone often led to over- or underpredictions, highlighting the important role of surface pH in the dissolution of weak bases such as entrectinib. Incorporating surface pH into *in silico* simulations is essential as current commercial software PBPK models do not include this calculation for weak bases with pH-modifying excipients. The proposed approach demonstrates the importance of surface pH in accurately predicting *in vivo* dissolution for these types of drugs and can be used to optimize the formulation screening process.

## Data Availability

The original contributions presented in the study are included in the article/Supplementary Material; further inquiries can be directed to the corresponding author.
