# Supplementary material for: Mechanistic mass transport analysis of dissolution of weak-base drugs considering the influence of surface pH in formulations with and without acidifying agents
Source: Front Pharmacol. 2026 Oct 1;17:1924679. doi: 10.3389/fphar.2026.1924679 (PMC13670172; doi:10.3389/fphar.2026.1924679)
Supplement: Supplementary file 1 [file Supplementaryfile1.docx]

**SUPPLEMENTARY MATERIAL**

**Mechanistic mass transport analysis of dissolution of weak base drugs considering the influence of surface pH in formulations with and without acidulant**

**S1: Theoretical surface pH prediction in presence of unbuffered media**

a) **Case 1:** **Mass transport equations for a dibasic drug with monoprotic acidulant (unbuffered)**
b) $D_{A}\left( \frac{D_{HA}[HA]_{h}+D_{A}[A]_{h}}{D_{A}+D_{HA}\left( \frac{[H^{+}]_{0}}{K_{a3}} \right)}-[A]_{h} \right)+D_{OH}\left( \frac{K_{w}}{[H^{+}]_{0}}-[O{H]}_{h} \right)=D_{BH}\left( \frac{[B]_{0}[H^{+}]_{0}}{K_{a1}}-[BH^{+}]_{h} \right)+2D_{BH_{2}^{2+}}\left( \frac{[B]_{0}{[H^{+}]_{0}}^{2}}{K_{a1}K_{a2}}-[BH_{2}^{2+}]_{h} \right)+D_{H}\left( \left[ H^{+} \right]_{0}-[H^{+}]_{h} \right)$**Case 2:** **Surface pH equations for a dibasic drug with diprotic acidulant (unbuffered)**

$$D_{HA}\left( \frac{D_{H_{2}A}[H_{2}A]_{h}+D_{HA}[HA]_{h}+D_{A}[A]_{h}}{D_{H_{2}A}\left( \frac{\left[ H^{+} \right]_{0}}{K_{a3}} \right)+D_{A}\left( \frac{K_{a4}}{\left[ H^{+} \right]_{0}} \right)+D_{HA}}-[HA]_{h} \right)+2D_{A}\left( \frac{K_{a4}}{\left[ H^{+} \right]_{0}}\left( \frac{D_{H_{2}A}[H_{2}A]_{h}+D_{HA}[HA]_{h}+D_{A}[A]_{h}}{D_{H_{2}A}\left( \frac{\left[ H^{+} \right]_{0}}{K_{a3}} \right)+D_{A}\left( \frac{K_{a4}}{\left[ H^{+} \right]_{0}} \right)+D_{HA}} \right)-[A]_{h} \right)+D_{OH}\left( \frac{K_{w}}{\left[ H^{+} \right]_{0}}-[O{H]}_{h} \right)=D_{BH}\left( \frac{[B]_{0}\left[ H^{+} \right]_{0}}{K_{a1}}-[BH^{+}]_{h} \right)+2D_{BH_{2}^{2+}}\left( \frac{[B]_{0}{\left[ H^{+} \right]_{0}}^{2}}{K_{a1}K_{a2}}-[BH_{2}^{2+}]_{h} \right)+D_{H}\left( \left[ H^{+} \right]_{0}-[H^{+}]_{h} \right)$$

**S2: Theoretical surface pH prediction in presence of buffered media (Acetate buffer, as a case example)**

c) **Case 2:** **Mass transport equations for a dibasic drug with monoprotic acidulant (acetate buffer)**

The chemical equilibria and reactions that take place when a dibasic drug, B, dissolves under acetate buffer (monoprotic buffer) conditions are as follows:

$B+H^{+}\underset{\leftrightarrow}{K_{a1}}BH^{+}$	1

$BH^{+}+H^{+}\underset{\leftrightarrow}{K_{a2}}BH_{2}^{2+}$	2

$HA\underset{\leftrightarrow}{K_{a3}}H^{+}+A^{-}$	3

$CH_{3}COOH+H_{2}O\underset{\leftrightarrow}{K_{b}}CH_{3}COO^{-}+H^{+}$	4

$H^{+}+OH^{-}\underset{\leftrightarrow}{K_{w}}H_{2}O$	5

The chemical equilibrium rate constant reactions for the above reactions are as follows:

$K_{a1}=\frac{\left[ B \right][H^{+}]}{[BH^{+}]}$	6

$K_{a2}=\frac{\left[ BH^{+} \right][H^{+}]}{[BH_{2}^{2+}]}$	7

$K_{a3}=\frac{[H^{+}][A^{-}]}{[HA]}$	8

$K_{b}=\frac{\left[ CH_{3}COO^{-} \right][H^{+}]}{[CH_{3}COOH]}$	9

$K_{w}=\left[ H^{+} \right][OH^{-}]$	10

The flux of all species across the diffusion layer includes both diffusion and chemical reactions occurring during dissolution. Using the simplified film model approach by Mooney et al., the differential equations that describe the diffusion and simultaneous chemical reactions of the individual species within the diffusion layer at steady state are defined as follows (1,2):

$\frac{\partial[BH_{2}^{2+}]}{\partial t}=D_{B}\frac{\partial^{2} [BH_{2}^{2+}]}{\partial x^{2}}+\phi_{1}=0$	11

$\frac{\partial[BH^{+}]}{\partial t}=D_{B}\frac{\partial^{2} [BH^{+}]}{\partial x^{2}}+\phi_{2}=0$	12

$\frac{\partial[B]}{\partial t}=D_{B}\frac{\partial^{2} [B]}{\partial x^{2}}+\phi_{3}=0$	13

$\frac{\partial[\mathrm{OH}^{-}]}{\partial t}=D_{\mathrm{OH}^{-}}\frac{\partial^{2} \left[ \mathrm{OH}^{-} \right]}{\partial x^{2}}+\phi_{4}=0$	14

$\frac{\partial[H^{+}]}{\partial t}=D_{H^{+}}\frac{\partial^{2} \left[ H^{+} \right]}{\partial x^{2}}+\phi_{5}=0$	15

$\frac{\partial[HA]}{\partial t}=D_{H_{2}A}\frac{\partial^{2} [\mathrm{HA}]}{\partial x^{2}}+\phi_{6}=0$	16

$\frac{\partial[A^{-}]}{\partial t}=D_{A^{-}}\frac{\partial^{2} \left[ A^{-} \right]}{\partial x^{2}}+\phi_{7}=0$	17

$\frac{\partial[CH_{3}COOH]}{\partial t}=D_{CH_{3}\mathrm{COOH}}\frac{\partial^{2} [CH_{3}COOH]}{\partial x^{2}}+\phi_{8}=0$	18

$\frac{\partial[CH_{3}COO^{-}]}{\partial t}=D_{B}\frac{\partial^{2} [CH_{3}COO^{-}]}{\partial x^{2}}+\phi_{9}=0$	19

Mass and charge balance considerations give the following relationships between $\phi_{i}$ terms:

At equilibrium, the reaction rate of the reactant should be the opposite of the product. Based on the chemical equilibria:

$\phi_{1}=-\phi_{2}-\phi_{3}$	20

$\phi_{6}=-\phi_{5}-\phi_{7}$	21

$\phi_{8}=-\phi_{9}$	22

The reaction rate of the acid and the base should be the same:

$\phi_{1}+\phi_{5}+\phi_{6}+\phi_{8}=\phi_{3}+\phi_{4}+\phi_{7}+\phi_{9}$	23

This equation ensures that the net change in charge due to the reactions is zero.

Based on the equations 20, 21, 22, and 23, the following mass balance equations can be written as:

$D_{BH_{2}^{2+}}\frac{d^{2}[BH_{2}^{2+}]}{d^{2}x}+D_{\mathrm{BH}^{+}}\frac{d^{2}[\mathrm{BH}^{+}]}{d^{2}x}+D_{B}\frac{d^{2}[B]}{d^{2}x}=0$	24

$D_{HA}\frac{d^{2}[HA]}{d^{2}x}+D_{A^{-}}\frac{d^{2}[A^{-}]}{d^{2}x}=0$	25

$D_{CH_{3}COOH}\frac{d^{2}[CH_{3}COOH]}{dx^{2}}+D_{CH_{3}COO^{-}}\frac{d^{2}[CH_{3}COO^{-}]}{dx^{2}}=0$	26

$D_{BH_{2}^{2+}}\frac{d^{2}[BH_{2}^{2+}]}{d^{2}x}+D_{H^{+}}\frac{d^{2}\left[ H^{+} \right]}{d^{2}x}+D_{HA}\frac{d^{2}[HA]}{d^{2}x}+D_{CH_{3}COOH}\frac{d^{2}[CH_{3}COOH]}{dx^{2}}-D_{\mathrm{OH}^{-}}\frac{d^{2}\left[ \mathrm{OH}^{-} \right]}{d^{2}x}-D_{B}\frac{d^{2}\left[ B \right]}{d^{2}x}-D_{A^{-}}\frac{d^{2}[A^{-}]}{d^{2}x}-D_{CH_{3}COO^{-}}\frac{d^{2}[CH_{3}COO^{-}]}{dx^{2}}=0$	27

Integrating above equations gives:

$D_{BH_{2}^{2+}}\frac{d[BH_{2}^{2+}]}{\mathrm{dx}}+D_{\mathrm{BH}^{+}}\frac{d[\mathrm{BH}^{+}]}{\mathrm{dx}}+D_{B}\frac{d[B]}{\mathrm{dx}}=C_{1}$	28

$D_{HA}\frac{d[HA]}{\mathrm{dx}}+D_{A^{-}}\frac{d[A^{-}]}{\mathrm{dx}}=C_{2}$	29

$D_{CH_{3}COOH}\frac{d[CH_{3}COOH]}{\mathrm{dx}}+D_{CH_{3}COO^{-}}\frac{d[CH_{3}COO^{-}]}{\mathrm{dx}}=C_{3}$	30

$D_{BH_{2}^{2+}}\frac{d[BH_{2}^{2+}]}{dx}+D_{H^{+}}\frac{d\left[ H^{+} \right]}{dx}+D_{HA}\frac{d[HA]}{\mathrm{dx}}+D_{CH_{3}COOH}\frac{d[CH_{3}COOH]}{\mathrm{dx}}-D_{\mathrm{OH}^{-}}\frac{d\left[ \mathrm{OH}^{-} \right]}{dx}-D_{B}\frac{d\left[ B \right]}{dx}-D_{A^{-}}\frac{d[A^{-}]}{\mathrm{dx}}-D_{CH_{3}COO^{-}}\frac{d[CH_{3}COO^{-}]}{\mathrm{dx}}=C_{4}$	31

According to Fick's first law of diffusion, the $D_{i}\frac{dC_{i}}{\mathrm{dt}}$terms in the equations above represent the fluxes of the respective species. By charge neutrality, the sum of the fluxes of species reacting as acids must equal the sum of the fluxes of species reacting as bases.

$\sum\left( J_{BH_{2}^{2+}}+J_{\mathrm{BH}^{+}}+J_{H^{+}}+J_{CH_{3}COOH} \right)=\sum\left( J_{\mathrm{OH}^{-}}+J_{A^{-}}+J_{CH_{3}COO^{-}} \right)$	32

Integrating the equations 28, 29, 30, and 31 with respect to x:

$D_{BH_{2}^{2+}}\left[ BH_{2}^{2+} \right]+D_{\mathrm{BH}^{+}}\left[ \mathrm{BH}^{+} \right]+D_{B}\left[ B \right]=C_{1}x+C_{5}$	33

$D_{HA}\left[ HA \right]+D_{A^{-}}\left[ A^{-} \right]=C_{2}x+C_{6}$	34

$D_{CH_{3}COOH}\left[ CH_{3}COOH \right]+D_{CH_{3}COO^{-}}\left[ CH_{3}COO^{-} \right]=C_{3}x+C_{7}$	35

$D_{BH_{2}^{2+}}\left[ BH_{2}^{2+} \right]+D_{H^{+}}\left[ H^{+} \right]+D_{HA}\left[ HA \right]+D_{CH_{3}COOH}\left[ CH_{3}COOH \right]-D_{\mathrm{OH}^{-}}\left[ \mathrm{OH}^{-} \right]-D_{B}\left[ B \right]-D_{A^{-}}\left[ A^{-} \right]-D_{CH_{3}COO^{-}}\left[ CH_{3}COO^{-} \right]=C_{4}x+C_{8}$	36

The boundary conditions for the differential equations are defined as:

At x = 0 (at the surface of the dissolving solid):

$$\left[ B \right]={[B]}_{0}(intrinsic solubility of the drug)$$

$$\left[ {BH}^{+} \right]=[{BH}^{+}]_{0} unknown$$

$$\left[ BH_{2}^{2+} \right]=[{BH_{2}^{2+}]}_{0} unknown$$

$$\left[ A^{-} \right]=\left[ A^{-} \right]_{0} unknown$$

$$\left[ CH_{3}COOH \right]=\left[ CH_{3}COOH \right]_{0} unknown$$

$$\left[ CH_{3}COO^{-} \right]=\left[ CH_{3}COO^{-} \right]_{0} unknown$$

$$\left[ H^{+} \right]=\left[ H^{+} \right]_{0} unknown$$

$$\left[ {OH}^{-} \right]=\left[ {OH}^{-} \right]_{0} unknown$$

where the subscript 0 represents the surface concentration of the respective species

At x = h (the boundary between the diffusion layer and the bulk medium)

$$\left[ B \right]={[B]}_{h}=0 (sink condition)$$

$$\left[ {BH}^{+} \right]=[{BH}^{+}]_{h}=0 (sink condition)$$

$$\left[ BH_{2}^{2+} \right]=[{BH_{2}^{2+}]}_{h}=0 (sink condtion)$$

$$\left[ A^{-} \right]=\left[ A^{-} \right]_{h}=0 (sink condtion)$$

$$\left[ CH_{3}COO^{-} \right]=[CH_{3}COO^{-}]_{h} known$$

$$\left[ CH_{3}COOH \right]=[CH_{3}COOH]_{h} known$$

$$\left[ H^{+} \right]=\left[ H^{+} \right]_{h} known$$

$$\left[ {OH}^{-} \right]=\left[ {OH}^{-} \right]_{h} known$$

where the subscript h represents the bulk concentration of the respective species

Applying the above boundary conditions to equation 33, 34, 35, and 36, at x = 0:

$D_{BH_{2}^{2+}}[BH_{2}^{2+}]_{0}+D_{\mathrm{BH}^{+}}[BH^{+}]_{0}+D_{B}[B]_{0}=C_{5}$	37

$D_{\mathrm{HA}}[HA]_{0}+D_{A^{-}}[A^{-}]_{0}=C_{6}$	38

$D_{CH_{3}COOH}[CH_{3}COOH]_{0}+D_{CH_{3}COO^{-}}[CH_{3}COO^{-}]_{0}=C_{7}$	39

$D_{BH_{2}^{2+}}[BH_{2}^{2+}]_{0}+D_{H^{+}}[H^{+}]_{0}+D_{\mathrm{HA}}[HA]_{0}+D_{CH_{3}COOH}[CH_{3}COOH]_{0}-D_{\mathrm{OH}^{-}}[OH^{-}]_{0}-D_{B}[B]_{0}-D_{A^{-}}[A^{-}]_{0}-D_{CH_{3}COO^{-}}[CH_{3}COO^{-}]_{0}=C_{8}$	40

Applying the above boundary conditions to equation 33, 34, 35, and 36, at x = h:

$0=C_{1}h+C_{5}$	41

$0=C_{2}h+C_{6}$	42

$D_{CH_{3}COOH}[CH_{3}COOH]_{h}+D_{CH_{3}COO^{-}}[CH_{3}COO^{-}]_{h}=C_{3}h+C_{7}$	43

$D_{H^{+}}[H^{+}]_{h}+D_{CH_{3}COOH}[CH_{3}COOH]_{h}-D_{\mathrm{OH}^{-}}[OH^{-}]_{h}-D_{CH_{3}COO^{-}}[CH_{3}COO^{-}]_{h}=C_{4}h+C_{8}$	44

Subtracting equations 37 and 41 results in

$D_{BH_{2}^{2+}}[BH_{2}^{2+}]_{0}+D_{\mathrm{BH}^{+}}[BH^{+}]_{0}+D_{B}[B]_{0}=-C_{1}h$	45

Subtracting equations 38 and 42 results in

$D_{HA}[HA]_{0}+D_{A^{-}}[A^{-}]_{0}=-C_{2}h$	46

Subtracting equations 39 and 43 results in

$D_{CH_{3}COOH}\left( [CH_{3}COOH]_{0}-[CH_{3}COOH]_{h} \right)+D_{CH_{3}COO^{-}}\left( [CH_{3}COO^{-}]_{0}-[CH_{3}COO^{-}]_{h} \right)=-C_{3}h$	47

Subtracting equations 40 and 44 results in

$D_{BH_{2}^{2+}}[BH_{2}^{2+}]_{0}+D_{\mathrm{HA}}[HA]_{0}+D_{CH_{3}COOH}\left( [CH_{3}COOH]_{0}-[CH_{3}COOH]_{h} \right)-D_{CH_{3}COO^{-}}\left( [CH_{3}COO^{-}]_{0}-[CH_{3}COO^{-}]_{h} \right)+D_{H^{+}}\left( [H^{+}]_{0}-[H^{+}]_{h} \right)-D_{\mathrm{OH}^{-}}\left( [OH^{-}]_{0}-[OH^{-}]_{h} \right)-D_{B}[B]_{0}-D_{A^{-}}[A^{-}]_{0}=-C_{4}h$	48

Combining equations 45, 46, 47, and 48 and successive substation to solve the equations for a single unknown term ($\left[ H^{+} \right]_{0}$), yields the following equation:

$D_{A}\left( \frac{D_{HA}[HA]_{h}+D_{A}[A]_{h}}{D_{A}+D_{HA}\left( \frac{\left[ H^{+} \right]_{0}}{K_{a3}} \right)}-[A]_{h} \right)+D_{OH}\left( \frac{K_{w}}{\left[ H^{+} \right]_{0}}-[O{H]}_{h} \right)+D_{CH_{3}COO^{-}}\left( \left( \frac{D_{CH_{3}COOH}[CH_{3}COO{H]}_{h}+D_{CH_{3}COO^{-}}[CH_{3}COO^{-}]_{h}}{D_{CH_{3}COOH}\left( \frac{\left[ H^{+} \right]_{0}}{K_{a4}} \right)+D_{CH_{3}COO^{-}}} \right)-[CH_{3}COO^{-}]_{h} \right)=D_{CH_{3}COOH}[CH_{3}COO{H]}_{h}+D_{BH}\left( \frac{[B]_{0}\left[ H^{+} \right]_{0}}{K_{a1}}-[BH^{+}]_{h} \right)+2D_{BH_{2}^{2+}}\left( \frac{[B]_{0}{\left[ H^{+} \right]_{0}}^{2}}{K_{a1}K_{a2}}-[BH_{2}^{2+}]_{h} \right)+D_{H}\left( \left[ H^{+} \right]_{0}-[H^{+}]_{h} \right)$	49

After cross multiplication with $\left[ H^{+} \right]_{0}$and rearranging above equation yields the final format of fourth power polynomial equation as a function of the concentration of hydrogen ion at the solid surface:

$p{\left[ H^{+} \right]_{0}}^{4}+q{\left[ H^{+} \right]_{0}}^{3}+r{\left[ H^{+} \right]_{0}}^{2}+s\left[ H^{+} \right]_{0}+t=0$	50

Where:

$$p=2D_{BH_{2}^{2+}}D_{HA}D_{CH_{3}COOH}\left[ B \right]_{0}$$

$$q=D_{HA}D_{CH_{3}COOH}D_{H^{+}}K_{a1}K_{a2}+D_{HA}D_{CH_{3}COOH}D_{BH}\left[ B \right]_{0}K_{a2}+2D_{A}D_{CH_{3}COOH}D_{BH_{2}^{2+}}\left[ B \right]_{0}K_{a3}$$

$$r=-2D_{HA}D_{CH_{3}COOH}D_{BH_{2}^{2+}}[BH_{2}^{2+}]_{h}K_{a1}K_{a2}+D_{A}D_{CH_{3}COOH}D_{H}K_{a1}K_{a2}K_{a3}+D_{HA}D_{CH_{3}COO^{-}}D_{CH_{3}COOH}[CH_{3}COO^{-}]_{h}K_{a1}K_{a2}+D_{A}D_{CH_{3}COOH}D_{BH}\left[ B \right]_{0}K_{a2}K_{a3}+D_{HA}D_{OH}D_{CH_{3}COOH}[O{H]}_{h}K_{a1}K_{a2}+D_{HA}D_{A}D_{CH_{3}COOH}[A]_{h}K_{a1}K_{a2}-D_{HA}D_{CH_{3}COOH}D_{H}[H^{+}]_{h}K_{a1}K_{a2}-D_{HA}D_{CH_{3}COOH}D_{BH}[BH^{+}]_{h}K_{a1}K_{a2}+D_{HA}D_{CH_{3}COOH}^{2}[CH_{3}COO{H]}_{h}K_{a1}K_{a2}-D_{HA}D_{CH_{3}COO^{-}}^{2}[CH_{3}COO^{-}]_{h}K_{a1}K_{a2}$$

$$s=-2D_{A}D_{CH_{3}COOH}D_{BH_{2}^{2+}}[BH_{2}^{2+}]_{h}K_{a1}K_{a2}K_{a3}+D_{A}D_{CH_{3}COOH}D_{CH_{3}COO^{-}}[CH_{3}COO^{-}]_{h}K_{a1}K_{a2}K_{a3}+D_{A}D_{CH_{3}COOH}D_{OH}[O{H]}_{h}K_{a1}K_{a2}K_{a3}-D_{A}D_{CH_{3}COOH}D_{H}[H^{+}]_{h}K_{a1}K_{a2}K_{a3}-D_{A}D_{CH_{3}COOH}D_{BH}[BH^{+}]_{h}K_{a1}K_{a2}K_{a3}-D_{HA}D_{CH_{3}COO^{-}}D_{CH_{3}COOH}[CH_{3}COO{H]}_{h}K_{a1}K_{a2}K_{a4}+D_{A}D_{CH_{3}COOH}^{2}[CH_{3}COOH]_{h}K_{a1}K_{a2}K_{a3}-D_{CH_{3}COOH}D_{A}D_{HA}[HA]_{h}K_{a1}K_{a2}K_{a3}-D_{HA}D_{CH_{3}COO^{-}}^{2}[CH_{3}COO^{-}]_{h}K_{a1}K_{a2}K_{a4}-D_{A}D_{CH_{3}COO^{-}}^{2}[CH_{3}COO^{-}]_{h}K_{a1}K_{a2}K_{a3}-D_{HA}D_{OH}D_{CH_{3}COOH}K_{a1}K_{a2}K_{w}$$

$$t=-D_{A}D_{CH_{3}COOH}D_{CH_{3}COO^{-}}[CH_{3}COOH]_{h}K_{a1}K_{a2}K_{a3}K_{a4}-D_{A}D_{CH_{3}COO^{-}}^{2}[CH_{3}COO^{-}]_{h}K_{a1}K_{a2}K_{a3}K_{a4}-D_{A}D_{OH}D_{CH_{3}COOH}K_{w}K_{a1}K_{a2}K_{a3}$$

The polynomial equation was numerically solved using the “roots” function of MATLAB software.

d) **Case 2:** **Surface pH equation for a dibasic drug with diprotic acidulant (acetate buffer)**

$$D_{HA}\left( \frac{D_{H_{2}A}[H_{2}A]_{h}+D_{HA}[HA]_{h}+D_{A}[A]_{h}}{D_{H_{2}A}\left( \frac{\left[ H^{+} \right]_{0}}{K_{a3}} \right)+D_{A}\left( \frac{K_{a4}}{\left[ H^{+} \right]_{0}} \right)+D_{HA}}-[HA]_{h} \right)+2D_{A}\left( \frac{K_{a4}}{\left[ H^{+} \right]_{0}}\left( \frac{D_{H_{2}A}[H_{2}A]_{h}+D_{HA}[HA]_{h}+D_{A}[A]_{h}}{D_{H_{2}A}\left( \frac{\left[ H^{+} \right]_{0}}{K_{a3}} \right)+D_{A}\left( \frac{K_{a4}}{\left[ H^{+} \right]_{0}} \right)+D_{HA}} \right)-[A]_{h} \right)+D_{OH}\left( \frac{K_{w}}{\left[ H^{+} \right]_{0}}-[O{H]}_{h} \right)+D_{CH_{3}COO^{-}}\left( \left( \frac{D_{CH_{3}COOH}[CH_{3}COO{H]}_{h}+D_{CH_{3}COO^{-}}[CH_{3}COO^{-}]_{h}}{D_{CH_{3}COOH}\left( \frac{\left[ H^{+} \right]_{0}}{K_{a5}} \right)+D_{CH_{3}COO^{-}}} \right)-[CH_{3}COO^{-}]_{h} \right)=D_{CH_{3}COOH}[CH_{3}COO{H]}_{h}+D_{BH}\left( \frac{[B]_{0}\left[ H^{+} \right]_{0}}{K_{a1}}-[BH^{+}]_{h} \right)+2D_{BH_{2}^{2+}}\left( \frac{[B]_{0}{\left[ H^{+} \right]_{0}}^{2}}{K_{a1}K_{a2}}-[BH_{2}^{2+}]_{h} \right)+D_{H}\left( \left[ H^{+} \right]_{0}-[H^{+}]_{h} \right)$$

Table S1: Key Input Data Used in the Surface pH Model

| **Description** | **Symbol** | **Value** | **Unit** |
| --- | --- | --- | --- |
| Diffusion coefficient; Water |  | 2.80E-05 | cm^2^/s |
| Diffusion coefficient; Buffer |  | 8.84E-06 | cm^2^/s |
| Diffusion coefficient; Entrectinib |  | 4.15E-07 | cm2/s |
| Diffusion coefficient; Betaine HCl (pH modifier) |  | 6.84E-07 | cm2/s |
| Diffusion coefficient; Tartaric acid (pH modifier) |  | 7.03E-07 | cm2/s |
| Buffer; Acetic acid | pKa5 | 4.6 |  |
| Dissociation constant; Buffer | K_a5_ | 2.51E-05 | M |
| Compound; Entrectinib | pKa1 | 2.54 |  |
| Compound; Entrectinib | pKa2 | 5.3 |  |
| Dissociation constant; Entrectinib | K_a1_ | 2.88E-03 | M |
| Dissociation constant; Entrectinib | K_a2_ | 5.01E-06 | M |
| Dissociation constant; water | k_w_ | 1.01E-14 | M |
| pH modifier; Betaine HCl | pKa3 | 1.94 |  |
| Dissociation constant; Betaine HCl (pH modifier) | K_a3_ | 0.011482 | M |
| pH modifier; Tartaric acid | pKa3 | 2.9 |  |
| pH modifier; Tartaric acid | pKa4 | 4.03 |  |
| Dissociation constant; Tartaric acid (pH modifier) | K_a3_ | 0.001259 | M |
| Dissociation constant; Tartaric acid (pH modifier) | K_a4_ | 9.33E-05 | M |
| Intrinsic solubility; Entrectinib |  | 2.14E-05 | M |

**(B)**

**(A)**

% Dissolved

| Time (h) | Reported | Predicted without surface pH | Predicted with surface pH |
| --- | --- | --- | --- |
| 0 | 0 | 0 | 0 |
| 0.00502513 |  | 0 | 0 |
| 0.0100503 |  | 0 | 0 |
| 0.0150754 |  | 0 | 0 |
| 0.0201005 |  | 0 | 0 |
| 0.0251256 |  | 0 | 0 |
| 0.0301508 |  | 0 | 0 |
| 0.0351759 |  | 71.4126 | 70.9039 |
| 0.040201 |  | 94.8381 | 94.7758 |
| 0.0452261 |  | 98.7176 | 98.7396 |
| 0.0502513 |  | 99.6563 | 99.6715 |
| 0.0552764 |  | 99.9047 | 99.9114 |
| 0.0603015 |  | 99.973 | 99.9756 |
| 0.0653266 |  | 99.9923 | 99.9932 |
| 0.0703518 |  | 99.9978 | 99.9981 |
| 0.0753769 |  | 99.9993 | 99.9995 |
| 0.080402 |  | 99.9998 | 99.9998 |
| 0.0854271 |  | 99.9999 | 100 |
| 0.0904523 |  | 100 | 100 |
| 0.0954774 |  | 100 | 100 |
| 0.100503 |  | 100 | 100 |
| 0.105528 |  | 100 | 100 |
| 0.110553 |  | 100 | 100 |
| 0.115578 |  | 100 | 100 |
| 0.120603 |  | 100 | 100 |
| 0.125628 |  | 100 | 100 |
| 0.130653 |  | 100 | 100 |
| 0.135678 |  | 100 | 100 |
| 0.140704 |  | 100 | 100 |
| 0.145729 |  | 100 | 100 |
| 0.150754 |  | 100 | 100 |
| 0.155779 |  | 100 | 100 |
| 0.160804 |  | 100 | 100 |
| 0.165829 |  | 100 | 100 |
| 0.170854 |  | 100 | 100 |
| 0.175879 |  | 100 | 100 |
| 0.180905 |  | 100 | 100 |
| 0.18593 |  | 100 | 100 |
| 0.190955 |  | 100 | 100 |
| 0.19598 |  | 100 | 100 |
| 0.201005 |  | 100 | 100 |
| 0.20603 |  | 100 | 100 |
| 0.211055 |  | 100 | 100 |
| 0.21608 |  | 100 | 100 |
| 0.221106 |  | 100 | 100 |
| 0.226131 |  | 100 | 100 |
| 0.231156 |  | 100 | 100 |
| 0.236181 |  | 100 | 100 |
| 0.241206 |  | 100 | 100 |
| 0.246231 |  | 100 | 100 |
| 0.25 | 99 | 100 | 100 |
| 0.251256 |  | 100 | 100 |
| 0.256281 |  | 100 | 100 |
| 0.261307 |  | 100 | 100 |
| 0.266332 |  | 100 | 100 |
| 0.271357 |  | 100 | 100 |
| 0.276382 |  | 100 | 100 |
| 0.281407 |  | 100 | 100 |
| 0.286432 |  | 100 | 100 |
| 0.291457 |  | 100 | 100 |
| 0.296482 |  | 100 | 100 |
| 0.301508 |  | 100 | 100 |
| 0.306533 |  | 100 | 100 |
| 0.311558 |  | 100 | 100 |
| 0.316583 |  | 100 | 100 |
| 0.321608 |  | 100 | 100 |
| 0.326633 |  | 100 | 100 |
| 0.33 | 99 | 100 | 100 |
| 0.331658 |  | 100 | 100 |
| 0.336683 |  | 100 | 100 |
| 0.341709 |  | 100 | 100 |
| 0.346734 |  | 100 | 100 |
| 0.351759 |  | 100 | 100 |
| 0.356784 |  | 100 | 100 |
| 0.361809 |  | 100 | 100 |
| 0.366834 |  | 100 | 100 |
| 0.371859 |  | 100 | 100 |
| 0.376884 |  | 100 | 100 |
| 0.38191 |  | 100 | 100 |
| 0.386935 |  | 100 | 100 |
| 0.39196 |  | 100 | 100 |
| 0.396985 |  | 100 | 100 |
| 0.40201 |  | 100 | 100 |
| 0.407035 |  | 100 | 100 |
| 0.41206 |  | 100 | 100 |
| 0.417085 |  | 100 | 100 |
| 0.422111 |  | 100 | 100 |
| 0.427136 |  | 100 | 100 |
| 0.432161 |  | 100 | 100 |
| 0.437186 |  | 100 | 100 |
| 0.442211 |  | 100 | 100 |
| 0.447236 |  | 100 | 100 |
| 0.452261 |  | 100 | 100 |
| 0.457286 |  | 100 | 100 |
| 0.462312 |  | 100 | 100 |
| 0.467337 |  | 100 | 100 |
| 0.472362 |  | 100 | 100 |
| 0.477387 |  | 100 | 100 |
| 0.482412 |  | 100 | 100 |
| 0.487437 |  | 100 | 100 |
| 0.492462 |  | 100 | 100 |
| 0.497487 |  | 100 | 100 |
| 0.5 | 99 | 100 | 100 |
| 0.502513 |  | 100 | 100 |
| 0.507538 |  | 100 | 100 |
| 0.512563 |  | 100 | 100 |
| 0.517588 |  | 100 | 100 |
| 0.522613 |  | 100 | 100 |
| 0.527638 |  | 100 | 100 |
| 0.532663 |  | 100 | 100 |
| 0.537688 |  | 100 | 100 |
| 0.542714 |  | 100 | 100 |
| 0.547739 |  | 100 | 100 |
| 0.552764 |  | 100 | 100 |
| 0.557789 |  | 100 | 100 |
| 0.562814 |  | 100 | 100 |
| 0.567839 |  | 100 | 100 |
| 0.572864 |  | 100 | 100 |
| 0.577889 |  | 100 | 100 |
| 0.582915 |  | 100 | 100 |
| 0.58794 |  | 100 | 100 |
| 0.592965 |  | 100 | 100 |
| 0.59799 |  | 100 | 100 |
| 0.603015 |  | 100 | 100 |
| 0.60804 |  | 100 | 100 |
| 0.613065 |  | 100 | 100 |
| 0.61809 |  | 100 | 100 |
| 0.623116 |  | 100 | 100 |
| 0.628141 |  | 100 | 100 |
| 0.633166 |  | 100 | 100 |
| 0.638191 |  | 100 | 100 |
| 0.643216 |  | 100 | 100 |
| 0.648241 |  | 100 | 100 |
| 0.653266 |  | 100 | 100 |
| 0.658291 |  | 100 | 100 |
| 0.663317 |  | 100 | 100 |
| 0.668342 |  | 100 | 100 |
| 0.67 | 99 | 100 | 100 |
| 0.673367 |  | 100 | 100 |
| 0.678392 |  | 100 | 100 |
| 0.683417 |  | 100 | 100 |
| 0.688442 |  | 100 | 100 |
| 0.693467 |  | 100 | 100 |
| 0.698492 |  | 100 | 100 |
| 0.703518 |  | 100 | 100 |
| 0.708543 |  | 100 | 100 |
| 0.713568 |  | 100 | 100 |
| 0.718593 |  | 100 | 100 |
| 0.723618 |  | 100 | 100 |
| 0.728643 |  | 100 | 100 |
| 0.733668 |  | 100 | 100 |
| 0.738693 |  | 100 | 100 |
| 0.743719 |  | 100 | 100 |
| 0.748744 |  | 100 | 100 |
| 0.75 | 99 | 100 | 100 |
| 0.753769 |  | 100 | 100 |
| 0.758794 |  | 100 | 100 |
| 0.763819 |  | 100 | 100 |
| 0.768844 |  | 100 | 100 |
| 0.773869 |  | 100 | 100 |
| 0.778894 |  | 100 | 100 |
| 0.78392 |  | 100 | 100 |
| 0.788945 |  | 100 | 100 |
| 0.79397 |  | 100 | 100 |
| 0.798995 |  | 100 | 100 |
| 0.80402 |  | 100 | 100 |
| 0.809045 |  | 100 | 100 |
| 0.81407 |  | 100 | 100 |
| 0.819095 |  | 100 | 100 |
| 0.824121 |  | 100 | 100 |
| 0.829146 |  | 100 | 100 |
| 0.834171 |  | 100 | 100 |
| 0.839196 |  | 100 | 100 |
| 0.844221 |  | 100 | 100 |
| 0.849246 |  | 100 | 100 |
| 0.854271 |  | 100 | 100 |
| 0.859296 |  | 100 | 100 |
| 0.864322 |  | 100 | 100 |
| 0.869347 |  | 100 | 100 |
| 0.874372 |  | 100 | 100 |
| 0.879397 |  | 100 | 100 |
| 0.884422 |  | 100 | 100 |
| 0.889447 |  | 100 | 100 |
| 0.894472 |  | 100 | 100 |
| 0.899497 |  | 100 | 100 |
| 0.904523 |  | 100 | 100 |
| 0.909548 |  | 100 | 100 |
| 0.914573 |  | 100 | 100 |
| 0.919598 |  | 100 | 100 |
| 0.924623 |  | 100 | 100 |
| 0.929648 |  | 100 | 100 |
| 0.934673 |  | 100 | 100 |
| 0.939698 |  | 100 | 100 |
| 0.944724 |  | 100 | 100 |
| 0.949749 |  | 100 | 100 |
| 0.954774 |  | 100 | 100 |
| 0.959799 |  | 100 | 100 |
| 0.964824 |  | 100 | 100 |
| 0.969849 |  | 100 | 100 |
| 0.974874 |  | 100 | 100 |

% Dissolved

| Time (h) | Reported | Predicted with out surface pH | Predicted with surface pH |
| --- | --- | --- | --- |
| 0 | 0 | 0 | 0 |
| 0.00502513 |  | 0 | 0 |
| 0.0100503 |  | 0 | 0 |
| 0.0150754 |  | 0 | 0 |
| 0.0201005 |  | 0 | 0 |
| 0.0251256 |  | 0 | 0 |
| 0.0301508 |  | 0 | 0 |
| 0.0351759 |  | 7.04811 | 0.607796 |
| 0.040201 |  | 21.3147 | 2.0787 |
| 0.0452261 |  | 30.247 | 3.31809 |
| 0.0502513 |  | 36.3256 | 4.36856 |
| 0.0552764 |  | 41.1908 | 5.26334 |
| 0.0603015 |  | 45.2248 | 6.02876 |
| 0.0653266 |  | 46.6436 | 6.68592 |
| 0.0703518 |  | 51.5924 | 7.25194 |
| 0.0753769 |  | 54.1724 | 7.74073 |
| 0.080402 |  | 54.1724 | 8.16385 |
| 0.0854271 |  | 54.1724 | 8.5309 |
| 0.0904523 |  | 54.1724 | 8.84989 |
| 0.0954774 |  | 54.1724 | 9.12753 |
| 0.100503 |  | 54.1724 | 9.36951 |
| 0.105528 |  | 54.1724 | 9.58068 |
| 0.110553 |  | 54.1724 | 9.76515 |
| 0.115578 |  | 54.1724 | 9.92642 |
| 0.120603 |  | 54.1724 | 10.0675 |
| 0.125628 |  | 54.1724 | 10.1911 |
| 0.130653 |  | 54.1724 | 10.2993 |
| 0.135678 |  | 54.1724 | 10.3943 |
| 0.140704 |  | 54.1724 | 10.4776 |
| 0.145729 |  | 54.1724 | 10.5507 |
| 0.150754 |  | 54.1724 | 10.6148 |
| 0.155779 |  | 54.1724 | 10.6711 |
| 0.160804 |  | 54.1724 | 10.7206 |
| 0.165829 |  | 54.1724 | 10.764 |
| 0.170854 |  | 54.1724 | 10.8022 |
| 0.175879 |  | 54.1724 | 10.8358 |
| 0.180905 |  | 54.1724 | 10.8653 |
| 0.18593 |  | 54.1724 | 10.8912 |
| 0.190955 |  | 54.1724 | 10.914 |
| 0.19598 |  | 54.1724 | 10.9341 |
| 0.201005 |  | 54.1724 | 10.9517 |
| 0.20603 |  | 54.1724 | 10.9672 |
| 0.211055 |  | 54.1724 | 10.9809 |
| 0.21608 |  | 54.1724 | 10.9929 |
| 0.221106 |  | 54.1724 | 11.0035 |
| 0.226131 |  | 54.1724 | 11.0128 |
| 0.231156 |  | 54.1724 | 11.0209 |
| 0.236181 |  | 54.1724 | 11.0281 |
| 0.241206 |  | 54.1724 | 11.0345 |
| 0.246231 |  | 54.1724 | 11.04 |
| 0.25 | 10 | 54.1724 | 11.0438 |
| 0.251256 |  | 54.1724 | 11.0449 |
| 0.256281 |  | 54.1724 | 11.0493 |
| 0.261307 |  | 54.1724 | 11.0531 |
| 0.266332 |  | 54.1724 | 11.0564 |
| 0.271357 |  | 54.1724 | 11.0593 |
| 0.276382 |  | 54.1724 | 11.0619 |
| 0.281407 |  | 54.1724 | 11.0642 |
| 0.286432 |  | 54.1724 | 11.0662 |
| 0.291457 |  | 54.1724 | 11.068 |
| 0.296482 |  | 54.1724 | 11.0695 |
| 0.301508 |  | 54.1724 | 11.0709 |
| 0.306533 |  | 54.1724 | 11.0721 |
| 0.311558 |  | 54.1724 | 11.0731 |
| 0.316583 |  | 54.1724 | 11.0741 |
| 0.321608 |  | 54.1724 | 11.0749 |
| 0.326633 |  | 54.1724 | 11.0756 |
| 0.33 | 12 | 54.1724 | 11.0761 |
| 0.331658 |  | 54.1724 | 11.0763 |
| 0.336683 |  | 54.1724 | 11.0768 |
| 0.341709 |  | 54.1724 | 11.0773 |
| 0.346734 |  | 54.1724 | 11.0777 |
| 0.351759 |  | 54.1724 | 11.0781 |
| 0.356784 |  | 54.1724 | 11.0785 |
| 0.361809 |  | 54.1724 | 11.0787 |
| 0.366834 |  | 54.1724 | 11.079 |
| 0.371859 |  | 54.1724 | 11.0792 |
| 0.376884 |  | 54.1724 | 11.0794 |
| 0.38191 |  | 54.1724 | 11.0796 |
| 0.386935 |  | 54.1724 | 11.0797 |
| 0.39196 |  | 54.1724 | 11.0799 |
| 0.396985 |  | 54.1724 | 11.08 |
| 0.40201 |  | 54.1724 | 11.0801 |
| 0.407035 |  | 54.1724 | 11.0802 |
| 0.41206 |  | 54.1724 | 11.0803 |
| 0.417085 |  | 54.1724 | 11.0804 |
| 0.422111 |  | 54.1724 | 11.0804 |
| 0.427136 |  | 54.1724 | 11.0805 |
| 0.432161 |  | 54.1724 | 11.0806 |
| 0.437186 |  | 54.1724 | 11.0806 |
| 0.442211 |  | 54.1724 | 11.0807 |
| 0.447236 |  | 54.1724 | 11.0807 |
| 0.452261 |  | 54.1724 | 11.0807 |
| 0.457286 |  | 54.1724 | 11.0807 |
| 0.462312 |  | 54.1724 | 11.0808 |
| 0.467337 |  | 54.1724 | 11.0808 |
| 0.472362 |  | 54.1724 | 11.0808 |
| 0.477387 |  | 54.1724 | 11.0808 |
| 0.482412 |  | 54.1724 | 11.0808 |
| 0.487437 |  | 54.1724 | 11.0808 |
| 0.492462 |  | 54.1724 | 11.0808 |
| 0.497487 |  | 54.1724 | 11.0809 |
| 0.5 | 11 | 54.1724 | 11.0809 |
| 0.502513 |  | 54.1724 | 11.0809 |
| 0.507538 |  | 54.1724 | 11.0809 |
| 0.512563 |  | 54.1724 | 11.0809 |
| 0.517588 |  | 54.1724 | 11.0809 |
| 0.522613 |  | 54.1724 | 11.0809 |
| 0.527638 |  | 54.1724 | 11.0809 |
| 0.532663 |  | 54.1724 | 11.0809 |
| 0.537688 |  | 54.1724 | 11.0809 |
| 0.542714 |  | 54.1724 | 11.0809 |
| 0.547739 |  | 54.1724 | 11.0809 |
| 0.552764 |  | 54.1724 | 11.0809 |
| 0.557789 |  | 54.1724 | 11.0809 |
| 0.562814 |  | 54.1724 | 11.0809 |
| 0.567839 |  | 54.1724 | 11.0809 |
| 0.572864 |  | 54.1724 | 11.0809 |
| 0.577889 |  | 54.1724 | 11.0809 |
| 0.582915 |  | 54.1724 | 11.0809 |
| 0.58794 |  | 54.1724 | 11.0809 |
| 0.592965 |  | 54.1724 | 11.0809 |
| 0.59799 |  | 54.1724 | 11.0809 |
| 0.603015 |  | 54.1724 | 11.0809 |
| 0.60804 |  | 54.1724 | 11.081 |
| 0.613065 |  | 54.1724 | 11.081 |
| 0.61809 |  | 54.1724 | 11.081 |
| 0.623116 |  | 54.1724 | 11.081 |
| 0.628141 |  | 54.1724 | 11.081 |
| 0.633166 |  | 54.1724 | 11.081 |
| 0.638191 |  | 54.1724 | 11.081 |
| 0.643216 |  | 54.1724 | 11.081 |
| 0.648241 |  | 54.1724 | 11.081 |
| 0.653266 |  | 54.1724 | 11.081 |
| 0.658291 |  | 54.1724 | 11.0809 |
| 0.663317 |  | 54.1724 | 11.0809 |
| 0.668342 |  | 54.1724 | 11.0809 |
| 0.67 | 11 | 54.1724 | 11.0809 |
| 0.673367 |  | 54.1724 | 11.0809 |
| 0.678392 |  | 54.1724 | 11.0809 |
| 0.683417 |  | 54.1724 | 11.0809 |
| 0.688442 |  | 54.1724 | 11.0809 |
| 0.693467 |  | 54.1724 | 11.081 |
| 0.698492 |  | 54.1724 | 11.081 |
| 0.703518 |  | 54.1724 | 11.081 |
| 0.708543 |  | 54.1724 | 11.081 |
| 0.713568 |  | 54.1724 | 11.081 |
| 0.718593 |  | 54.1724 | 11.081 |
| 0.723618 |  | 54.1724 | 11.081 |
| 0.728643 |  | 54.1724 | 11.081 |
| 0.733668 |  | 54.1724 | 11.081 |
| 0.738693 |  | 54.1724 | 11.081 |
| 0.743719 |  | 54.1724 | 11.081 |
| 0.748744 |  | 54.1724 | 11.081 |
| 0.75 | 11 | 54.1724 | 11.081 |
| 0.753769 |  | 54.1724 | 11.081 |
| 0.758794 |  | 54.1724 | 11.0809 |
| 0.763819 |  | 54.1724 | 11.0809 |
| 0.768844 |  | 54.1724 | 11.0809 |
| 0.773869 |  | 54.1724 | 11.0809 |
| 0.778894 |  | 54.1724 | 11.0809 |
| 0.78392 |  | 54.1724 | 11.0809 |
| 0.788945 |  | 54.1724 | 11.0809 |
| 0.79397 |  | 54.1724 | 11.0809 |
| 0.798995 |  | 54.1724 | 11.0809 |
| 0.80402 |  | 54.1724 | 11.0809 |
| 0.809045 |  | 54.1724 | 11.0809 |
| 0.81407 |  | 54.1724 | 11.0809 |
| 0.819095 |  | 54.1724 | 11.081 |
| 0.824121 |  | 54.1724 | 11.081 |
| 0.829146 |  | 54.1724 | 11.081 |
| 0.834171 |  | 54.1724 | 11.081 |
| 0.839196 |  | 54.1724 | 11.081 |
| 0.844221 |  | 54.1724 | 11.081 |
| 0.849246 |  | 54.1724 | 11.081 |
| 0.854271 |  | 54.1724 | 11.081 |
| 0.859296 |  | 54.1724 | 11.081 |
| 0.864322 |  | 54.1724 | 11.081 |
| 0.869347 |  | 54.1724 | 11.081 |
| 0.874372 |  | 54.1724 | 11.081 |
| 0.879397 |  | 54.1724 | 11.081 |
| 0.884422 |  | 54.1724 | 11.081 |
| 0.889447 |  | 54.1724 | 11.081 |
| 0.894472 |  | 54.1724 | 11.081 |
| 0.899497 |  | 54.1724 | 11.081 |
| 0.904523 |  | 54.1724 | 11.081 |
| 0.909548 |  | 54.1724 | 11.081 |
| 0.914573 |  | 54.1724 | 11.081 |
| 0.919598 |  | 54.1724 | 11.081 |
| 0.924623 |  | 54.1724 | 11.081 |
| 0.929648 |  | 54.1724 | 11.081 |
| 0.934673 |  | 54.1724 | 11.081 |
| 0.939698 |  | 54.1724 | 11.081 |
| 0.944724 |  | 54.1724 | 11.081 |
| 0.949749 |  | 54.1724 | 11.081 |
| 0.954774 |  | 54.1724 | 11.081 |
| 0.959799 |  | 54.1724 | 11.081 |
| 0.964824 |  | 54.1724 | 11.081 |
| 0.969849 |  | 54.1724 | 11.081 |
| 0.974874 |  | 54.1724 | 11.081 |

**Figure S1:** Predicted dissolution profiles using DDD Plus for F0 formulation (without acidifying agent) with (A) 0.1 M HCl pH 1 media and (B) 0.0001M HCl pH 4 unbuffered media. The dashed black line represents the model prediction using bulk pH, while the solid blue corresponds the prediction using surface pH, and filled blue circles denote the observed data.

**(B)**

**(A)**

% Dissolved

| Time (h) | Reported | Predicted without surface pH | Predicted with surface pH |
| --- | --- | --- | --- |
| 0 | 0 | 0 | 0 |
| 0.00502513 |  | 0 | 0 |
| 0.0100503 |  | 0 | 0 |
| 0.0150754 |  | 0 | 0 |
| 0.0201005 |  | 0 | 0 |
| 0.0251256 |  | 0 | 0 |
| 0.0301508 |  | 0 | 0 |
| 0.0351759 |  | 50.8238 | 50.1462 |
| 0.040201 |  | 92.0205 | 91.9327 |
| 0.0452261 |  | 95.4747 | 95.5001 |
| 0.0502513 |  | 97.0908 | 97.1399 |
| 0.0552764 |  | 98.2674 | 98.3234 |
| 0.0603015 |  | 99.0744 | 99.125 |
| 0.0653266 |  | 99.5814 | 99.6191 |
| 0.0703518 |  | 99.8582 | 99.8799 |
| 0.0753769 |  | 99.9744 | 99.9821 |
| 0.080402 |  | 99.9997 | 100 |
| 0.0854271 |  | 100 | 100 |
| 0.0904523 |  | 100 | 100 |
| 0.0954774 |  | 100 | 100 |
| 0.100503 |  | 100 | 100 |
| 0.105528 |  | 100 | 100 |
| 0.110553 |  | 100 | 100 |
| 0.115578 |  | 100 | 100 |
| 0.120603 |  | 100 | 100 |
| 0.125628 |  | 100 | 100 |
| 0.130653 |  | 100 | 100 |
| 0.135678 |  | 100 | 100 |
| 0.140704 |  | 100 | 100 |
| 0.145729 |  | 100 | 100 |
| 0.150754 |  | 100 | 100 |
| 0.155779 |  | 100 | 100 |
| 0.160804 |  | 100 | 100 |
| 0.165829 |  | 100 | 100 |
| 0.170854 |  | 100 | 100 |
| 0.175879 |  | 100 | 100 |
| 0.180905 |  | 100 | 100 |
| 0.18593 |  | 100 | 100 |
| 0.190955 |  | 100 | 100 |
| 0.19598 |  | 100 | 100 |
| 0.201005 |  | 100 | 100 |
| 0.20603 |  | 100 | 100 |
| 0.211055 |  | 100 | 100 |
| 0.21608 |  | 100 | 100 |
| 0.221106 |  | 100 | 100 |
| 0.226131 |  | 100 | 100 |
| 0.231156 |  | 100 | 100 |
| 0.236181 |  | 100 | 100 |
| 0.241206 |  | 100 | 100 |
| 0.246231 |  | 100 | 100 |
| 0.25 | 100 | 100 | 100 |
| 0.251256 |  | 100 | 100 |
| 0.256281 |  | 100 | 100 |
| 0.261307 |  | 100 | 100 |
| 0.266332 |  | 100 | 100 |
| 0.271357 |  | 100 | 100 |
| 0.276382 |  | 100 | 100 |
| 0.281407 |  | 100 | 100 |
| 0.286432 |  | 100 | 100 |
| 0.291457 |  | 100 | 100 |
| 0.296482 |  | 100 | 100 |
| 0.301508 |  | 100 | 100 |
| 0.306533 |  | 100 | 100 |
| 0.311558 |  | 100 | 100 |
| 0.316583 |  | 100 | 100 |
| 0.321608 |  | 100 | 100 |
| 0.326633 |  | 100 | 100 |
| 0.33 | 100 | 100 | 100 |
| 0.331658 |  | 100 | 100 |
| 0.336683 |  | 100 | 100 |
| 0.341709 |  | 100 | 100 |
| 0.346734 |  | 100 | 100 |
| 0.351759 |  | 100 | 100 |
| 0.356784 |  | 100 | 100 |
| 0.361809 |  | 100 | 100 |
| 0.366834 |  | 100 | 100 |
| 0.371859 |  | 100 | 100 |
| 0.376884 |  | 100 | 100 |
| 0.38191 |  | 100 | 100 |
| 0.386935 |  | 100 | 100 |
| 0.39196 |  | 100 | 100 |
| 0.396985 |  | 100 | 100 |
| 0.40201 |  | 100 | 100 |
| 0.407035 |  | 100 | 100 |
| 0.41206 |  | 100 | 100 |
| 0.417085 |  | 100 | 100 |
| 0.422111 |  | 100 | 100 |
| 0.427136 |  | 100 | 100 |
| 0.432161 |  | 100 | 100 |
| 0.437186 |  | 100 | 100 |
| 0.442211 |  | 100 | 100 |
| 0.447236 |  | 100 | 100 |
| 0.452261 |  | 100 | 100 |
| 0.457286 |  | 100 | 100 |
| 0.462312 |  | 100 | 100 |
| 0.467337 |  | 100 | 100 |
| 0.472362 |  | 100 | 100 |
| 0.477387 |  | 100 | 100 |
| 0.482412 |  | 100 | 100 |
| 0.487437 |  | 100 | 100 |
| 0.492462 |  | 100 | 100 |
| 0.497487 |  | 100 | 100 |
| 0.5 | 100 | 100 | 100 |
| 0.502513 |  | 100 | 100 |
| 0.507538 |  | 100 | 100 |
| 0.512563 |  | 100 | 100 |
| 0.517588 |  | 100 | 100 |
| 0.522613 |  | 100 | 100 |
| 0.527638 |  | 100 | 100 |
| 0.532663 |  | 100 | 100 |
| 0.537688 |  | 100 | 100 |
| 0.542714 |  | 100 | 100 |
| 0.547739 |  | 100 | 100 |
| 0.552764 |  | 100 | 100 |
| 0.557789 |  | 100 | 100 |
| 0.562814 |  | 100 | 100 |
| 0.567839 |  | 100 | 100 |
| 0.572864 |  | 100 | 100 |
| 0.577889 |  | 100 | 100 |
| 0.582915 |  | 100 | 100 |
| 0.58794 |  | 100 | 100 |
| 0.592965 |  | 100 | 100 |
| 0.59799 |  | 100 | 100 |
| 0.603015 |  | 100 | 100 |
| 0.60804 |  | 100 | 100 |
| 0.613065 |  | 100 | 100 |
| 0.61809 |  | 100 | 100 |
| 0.623116 |  | 100 | 100 |
| 0.628141 |  | 100 | 100 |
| 0.633166 |  | 100 | 100 |
| 0.638191 |  | 100 | 100 |
| 0.643216 |  | 100 | 100 |
| 0.648241 |  | 100 | 100 |
| 0.653266 |  | 100 | 100 |
| 0.658291 |  | 100 | 100 |
| 0.663317 |  | 100 | 100 |
| 0.668342 |  | 100 | 100 |
| 0.67 | 100 | 100 | 100 |
| 0.673367 |  | 100 | 100 |
| 0.678392 |  | 100 | 100 |
| 0.683417 |  | 100 | 100 |
| 0.688442 |  | 100 | 100 |
| 0.693467 |  | 100 | 100 |
| 0.698492 |  | 100 | 100 |
| 0.703518 |  | 100 | 100 |
| 0.708543 |  | 100 | 100 |
| 0.713568 |  | 100 | 100 |
| 0.718593 |  | 100 | 100 |
| 0.723618 |  | 100 | 100 |
| 0.728643 |  | 100 | 100 |
| 0.733668 |  | 100 | 100 |
| 0.738693 |  | 100 | 100 |
| 0.743719 |  | 100 | 100 |
| 0.748744 |  | 100 | 100 |
| 0.75 | 100 | 100 | 100 |
| 0.753769 |  | 100 | 100 |
| 0.758794 |  | 100 | 100 |
| 0.763819 |  | 100 | 100 |
| 0.768844 |  | 100 | 100 |
| 0.773869 |  | 100 | 100 |
| 0.778894 |  | 100 | 100 |
| 0.78392 |  | 100 | 100 |
| 0.788945 |  | 100 | 100 |
| 0.79397 |  | 100 | 100 |
| 0.798995 |  | 100 | 100 |
| 0.80402 |  | 100 | 100 |
| 0.809045 |  | 100 | 100 |
| 0.81407 |  | 100 | 100 |
| 0.819095 |  | 100 | 100 |
| 0.824121 |  | 100 | 100 |
| 0.829146 |  | 100 | 100 |
| 0.834171 |  | 100 | 100 |
| 0.839196 |  | 100 | 100 |
| 0.844221 |  | 100 | 100 |
| 0.849246 |  | 100 | 100 |
| 0.854271 |  | 100 | 100 |
| 0.859296 |  | 100 | 100 |
| 0.864322 |  | 100 | 100 |
| 0.869347 |  | 100 | 100 |
| 0.874372 |  | 100 | 100 |
| 0.879397 |  | 100 | 100 |
| 0.884422 |  | 100 | 100 |
| 0.889447 |  | 100 | 100 |
| 0.894472 |  | 100 | 100 |
| 0.899497 |  | 100 | 100 |
| 0.904523 |  | 100 | 100 |
| 0.909548 |  | 100 | 100 |
| 0.914573 |  | 100 | 100 |
| 0.919598 |  | 100 | 100 |
| 0.924623 |  | 100 | 100 |
| 0.929648 |  | 100 | 100 |
| 0.934673 |  | 100 | 100 |
| 0.939698 |  | 100 | 100 |
| 0.944724 |  | 100 | 100 |
| 0.949749 |  | 100 | 100 |
| 0.954774 |  | 100 | 100 |
| 0.959799 |  | 100 | 100 |
| 0.964824 |  | 100 | 100 |
| 0.969849 |  | 100 | 100 |
| 0.974874 |  | 100 | 100 |

% Dissolved

| Time (h) | Reported | Predicted with out surface pH | Predicted with surface pH |
| --- | --- | --- | --- |
| 0 | 0 | 0 | 0 |
| 0.00502513 |  | 0 | 0 |
| 0.0100503 |  | 0 | 0 |
| 0.0150754 |  | 0 | 0 |
| 0.0201005 |  | 0 | 0 |
| 0.0251256 |  | 0 | 0 |
| 0.0301508 |  | 0 | 0 |
| 0.0351759 |  | 7.04811 | 16.2727 |
| 0.040201 |  | 21.3147 | 29.3716 |
| 0.0452261 |  | 30.247 | 40.1064 |
| 0.0502513 |  | 36.3256 | 48.9936 |
| 0.0552764 |  | 41.1908 | 56.4071 |
| 0.0603015 |  | 45.2248 | 62.6243 |
| 0.0653266 |  | 46.6436 | 67.8545 |
| 0.0703518 |  | 51.5924 | 72.2592 |
| 0.0753769 |  | 54.1724 | 75.9649 |
| 0.080402 |  | 54.1724 | 79.0728 |
| 0.0854271 |  | 54.1724 | 81.6647 |
| 0.0904523 |  | 54.1724 | 83.8086 |
| 0.0954774 |  | 54.1724 | 85.562 |
| 0.100503 |  | 54.1724 | 86.9745 |
| 0.105528 |  | 54.1724 | 88.09 |
| 0.110553 |  | 54.1724 | 88.9486 |
| 0.115578 |  | 54.1724 | 89.5875 |
| 0.120603 |  | 54.1724 | 90.0422 |
| 0.125628 |  | 54.1724 | 90.3475 |
| 0.130653 |  | 54.1724 | 90.5381 |
| 0.135678 |  | 54.1724 | 90.649 |
| 0.140704 |  | 54.1724 | 90.7159 |
| 0.145729 |  | 54.1724 | 90.7724 |
| 0.150754 |  | 54.1724 | 90.8288 |
| 0.155779 |  | 54.1724 | 90.8851 |
| 0.160804 |  | 54.1724 | 90.9414 |
| 0.165829 |  | 54.1724 | 90.9976 |
| 0.170854 |  | 54.1724 | 91.0537 |
| 0.175879 |  | 54.1724 | 91.1096 |
| 0.180905 |  | 54.1724 | 91.1655 |
| 0.18593 |  | 54.1724 | 91.2212 |
| 0.190955 |  | 54.1724 | 91.2767 |
| 0.19598 |  | 54.1724 | 91.3321 |
| 0.201005 |  | 54.1724 | 91.3873 |
| 0.20603 |  | 54.1724 | 91.4422 |
| 0.211055 |  | 54.1724 | 91.497 |
| 0.21608 |  | 54.1724 | 91.5516 |
| 0.221106 |  | 54.1724 | 91.606 |
| 0.226131 |  | 54.1724 | 91.6601 |
| 0.231156 |  | 54.1724 | 91.7141 |
| 0.236181 |  | 54.1724 | 91.7678 |
| 0.241206 |  | 54.1724 | 91.8212 |
| 0.246231 |  | 54.1724 | 91.8744 |
| 0.25 | 89 | 54.1724 | 91.9142 |
| 0.251256 |  | 54.1724 | 91.9274 |
| 0.256281 |  | 54.1724 | 91.9802 |
| 0.261307 |  | 54.1724 | 92.0327 |
| 0.266332 |  | 54.1724 | 92.085 |
| 0.271357 |  | 54.1724 | 92.137 |
| 0.276382 |  | 54.1724 | 92.1888 |
| 0.281407 |  | 54.1724 | 92.2403 |
| 0.286432 |  | 54.1724 | 92.2916 |
| 0.291457 |  | 54.1724 | 92.3426 |
| 0.296482 |  | 54.1724 | 92.3934 |
| 0.301508 |  | 54.1724 | 92.4439 |
| 0.306533 |  | 54.1724 | 92.4942 |
| 0.311558 |  | 54.1724 | 92.5443 |
| 0.316583 |  | 54.1724 | 92.5941 |
| 0.321608 |  | 54.1724 | 92.6437 |
| 0.326633 |  | 54.1724 | 92.693 |
| 0.33 | 91 | 54.1724 | 92.742 |
| 0.331658 |  | 54.1724 | 92.7584 |
| 0.336683 |  | 54.1724 | 92.7909 |
| 0.341709 |  | 54.1724 | 92.8395 |
| 0.346734 |  | 54.1724 | 92.8878 |
| 0.351759 |  | 54.1724 | 92.9359 |
| 0.356784 |  | 54.1724 | 92.9838 |
| 0.361809 |  | 54.1724 | 93.0314 |
| 0.366834 |  | 54.1724 | 93.0788 |
| 0.371859 |  | 54.1724 | 93.126 |
| 0.376884 |  | 54.1724 | 93.1729 |
| 0.38191 |  | 54.1724 | 93.2196 |
| 0.386935 |  | 54.1724 | 93.2661 |
| 0.39196 |  | 54.1724 | 93.3123 |
| 0.396985 |  | 54.1724 | 93.3583 |
| 0.40201 |  | 54.1724 | 93.404 |
| 0.407035 |  | 54.1724 | 93.4496 |
| 0.41206 |  | 54.1724 | 93.4949 |
| 0.417085 |  | 54.1724 | 93.5399 |
| 0.422111 |  | 54.1724 | 93.5848 |
| 0.427136 |  | 54.1724 | 93.6294 |
| 0.432161 |  | 54.1724 | 93.6738 |
| 0.437186 |  | 54.1724 | 93.718 |
| 0.442211 |  | 54.1724 | 93.7619 |
| 0.447236 |  | 54.1724 | 93.8056 |
| 0.452261 |  | 54.1724 | 93.8491 |
| 0.457286 |  | 54.1724 | 93.8924 |
| 0.462312 |  | 54.1724 | 93.9354 |
| 0.467337 |  | 54.1724 | 93.9783 |
| 0.472362 |  | 54.1724 | 94.0209 |
| 0.477387 |  | 54.1724 | 94.0633 |
| 0.482412 |  | 54.1724 | 94.1055 |
| 0.487437 |  | 54.1724 | 94.1474 |
| 0.492462 |  | 54.1724 | 94.1892 |
| 0.497487 |  | 54.1724 | 94.2307 |
| 0.5 | 94 | 54.1724 | 94.2514 |
| 0.502513 |  | 54.1724 | 94.272 |
| 0.507538 |  | 54.1724 | 94.3131 |
| 0.512563 |  | 54.1724 | 94.354 |
| 0.517588 |  | 54.1724 | 94.3947 |
| 0.522613 |  | 54.1724 | 94.4351 |
| 0.527638 |  | 54.1724 | 94.4754 |
| 0.532663 |  | 54.1724 | 94.5154 |
| 0.537688 |  | 54.1724 | 94.5553 |
| 0.542714 |  | 54.1724 | 94.5949 |
| 0.547739 |  | 54.1724 | 94.6343 |
| 0.552764 |  | 54.1724 | 94.6735 |
| 0.557789 |  | 54.1724 | 94.7125 |
| 0.562814 |  | 54.1724 | 94.7513 |
| 0.567839 |  | 54.1724 | 94.7899 |
| 0.572864 |  | 54.1724 | 94.8283 |
| 0.577889 |  | 54.1724 | 94.8665 |
| 0.582915 |  | 54.1724 | 94.9045 |
| 0.58794 |  | 54.1724 | 94.9423 |
| 0.592965 |  | 54.1724 | 94.9799 |
| 0.59799 |  | 54.1724 | 95.0172 |
| 0.603015 |  | 54.1724 | 95.0544 |
| 0.60804 |  | 54.1724 | 95.0914 |
| 0.613065 |  | 54.1724 | 95.1282 |
| 0.61809 |  | 54.1724 | 95.1648 |
| 0.623116 |  | 54.1724 | 95.2012 |
| 0.628141 |  | 54.1724 | 95.2374 |
| 0.633166 |  | 54.1724 | 95.2734 |
| 0.638191 |  | 54.1724 | 95.3092 |
| 0.643216 |  | 54.1724 | 95.3448 |
| 0.648241 |  | 54.1724 | 95.3802 |
| 0.653266 |  | 54.1724 | 95.4155 |
| 0.658291 |  | 54.1724 | 95.4505 |
| 0.663317 |  | 54.1724 | 95.4854 |
| 0.668342 |  | 54.1724 | 95.5085 |
| 0.67 | 95 | 54.1724 | 95.52 |
| 0.673367 |  | 54.1724 | 95.5545 |
| 0.678392 |  | 54.1724 | 95.5888 |
| 0.683417 |  | 54.1724 | 95.6229 |
| 0.688442 |  | 54.1724 | 95.6568 |
| 0.693467 |  | 54.1724 | 95.6905 |
| 0.698492 |  | 54.1724 | 95.724 |
| 0.703518 |  | 54.1724 | 95.7574 |
| 0.708543 |  | 54.1724 | 95.7905 |
| 0.713568 |  | 54.1724 | 95.8235 |
| 0.718593 |  | 54.1724 | 95.8563 |
| 0.723618 |  | 54.1724 | 95.8889 |
| 0.728643 |  | 54.1724 | 95.9214 |
| 0.733668 |  | 54.1724 | 95.9536 |
| 0.738693 |  | 54.1724 | 95.9857 |
| 0.743719 |  | 54.1724 | 96.0176 |
| 0.748744 |  | 54.1724 | 96.0493 |
| 0.75 | 95 | 54.1724 | 96.0572 |
| 0.753769 |  | 54.1724 | 96.0808 |
| 0.758794 |  | 54.1724 | 96.1122 |
| 0.763819 |  | 54.1724 | 96.1434 |
| 0.768844 |  | 54.1724 | 96.1744 |
| 0.773869 |  | 54.1724 | 96.2052 |
| 0.778894 |  | 54.1724 | 96.2358 |
| 0.78392 |  | 54.1724 | 96.2663 |
| 0.788945 |  | 54.1724 | 96.2966 |
| 0.79397 |  | 54.1724 | 96.3268 |
| 0.798995 |  | 54.1724 | 96.3567 |
| 0.80402 |  | 54.1724 | 96.3865 |
| 0.809045 |  | 54.1724 | 96.4161 |
| 0.81407 |  | 54.1724 | 96.4456 |
| 0.819095 |  | 54.1724 | 96.4748 |
| 0.824121 |  | 54.1724 | 96.5039 |
| 0.829146 |  | 54.1724 | 96.5329 |
| 0.834171 |  | 54.1724 | 96.5616 |
| 0.839196 |  | 54.1724 | 96.5902 |
| 0.844221 |  | 54.1724 | 96.6187 |
| 0.849246 |  | 54.1724 | 96.6469 |
| 0.854271 |  | 54.1724 | 96.675 |
| 0.859296 |  | 54.1724 | 96.703 |
| 0.864322 |  | 54.1724 | 96.7307 |
| 0.869347 |  | 54.1724 | 96.7583 |
| 0.874372 |  | 54.1724 | 96.7858 |
| 0.879397 |  | 54.1724 | 96.8131 |
| 0.884422 |  | 54.1724 | 96.8402 |
| 0.889447 |  | 54.1724 | 96.8672 |
| 0.894472 |  | 54.1724 | 96.8939 |
| 0.899497 |  | 54.1724 | 96.9206 |
| 0.904523 |  | 54.1724 | 96.9471 |
| 0.909548 |  | 54.1724 | 96.9734 |
| 0.914573 |  | 54.1724 | 96.9995 |
| 0.919598 |  | 54.1724 | 97.0255 |
| 0.924623 |  | 54.1724 | 97.0514 |
| 0.929648 |  | 54.1724 | 97.0771 |
| 0.934673 |  | 54.1724 | 97.1026 |
| 0.939698 |  | 54.1724 | 97.128 |
| 0.944724 |  | 54.1724 | 97.1532 |
| 0.949749 |  | 54.1724 | 97.1783 |
| 0.954774 |  | 54.1724 | 97.2032 |
| 0.959799 |  | 54.1724 | 97.228 |
| 0.964824 |  | 54.1724 | 97.2526 |
| 0.969849 |  | 54.1724 | 97.277 |
| 0.974874 |  | 54.1724 | 97.3013 |

**Figure S2:** Predicted dissolution profiles using DDD Plus for F1 formulation (with monoprotic acidifying agent) were evaluated in unbuffered media: (A) 0.1 M HCl, pH 1; (B) 0.0001 M HCl, pH 4. The dashed black line represents the model prediction using bulk pH, while the solid blue corresponds the prediction using surface pH, and filled blue circles denotes the observed data

**(B)**

**(A)**

% Dissolved

| Time (h) | Reported | Predicted without surface pH | Predicted with surface pH |
| --- | --- | --- | --- |
| 0 | 0 | 0 | 0 |
| 0.00502513 |  | 0 | 0 |
| 0.0100503 |  | 0 | 0 |
| 0.0150754 |  | 0 | 0 |
| 0.0201005 |  | 0 | 0 |
| 0.0251256 |  | 0 | 0 |
| 0.0301508 |  | 0 | 0 |
| 0.0351759 |  | 0 | 0 |
| 0.040201 |  | 0 | 0 |
| 0.0452261 |  | 0 | 0 |
| 0.0502513 |  | 0 | 0 |
| 0.0552764 |  | 0 | 0 |
| 0.0603015 |  | 0 | 0 |
| 0.0653266 |  | 0 | 0 |
| 0.0703518 |  | 0 | 0 |
| 0.0753769 |  | 0 | 0 |
| 0.080402 |  | 0 | 0 |
| 0.0854271 |  | 0 | 0 |
| 0.0904523 |  | 0 | 0 |
| 0.0954774 |  | 0 | 0 |
| 0.100503 |  | 0 | 0 |
| 0.105528 |  | 0 | 0 |
| 0.110553 |  | 0 | 0 |
| 0.115578 |  | 0 | 0 |
| 0.120603 |  | 0 | 0 |
| 0.125628 |  | 0 | 0 |
| 0.130653 |  | 0 | 0 |
| 0.135678 |  | 0 | 0 |
| 0.140704 |  | 0 | 0 |
| 0.145729 |  | 0 | 0 |
| 0.150754 |  | 0 | 0 |
| 0.155779 |  | 0 | 0 |
| 0.160804 |  | 0 | 0 |
| 0.165829 |  | 0 | 0 |
| 0.170854 |  | 0 | 0 |
| 0.175879 |  | 0 | 0 |
| 0.180905 |  | 0 | 0 |
| 0.18593 |  | 0 | 0 |
| 0.190955 |  | 0 | 0 |
| 0.19598 |  | 0 | 0 |
| 0.201005 |  | 0 | 0 |
| 0.20603 |  | 0 | 0 |
| 0.211055 |  | 0 | 0 |
| 0.21608 |  | 0 | 0 |
| 0.221106 |  | 0 | 0 |
| 0.226131 |  | 0 | 0 |
| 0.231156 |  | 0 | 0 |
| 0.236181 |  | 72.6147 | 75.4777 |
| 0.241206 |  | 87.6341 | 92.9499 |
| 0.246231 |  | 91.8938 | 94.7548 |
| 0.25 | 17 | 94.1608 | 95.8847 |
| 0.251256 |  | 94.7813 | 96.2216 |
| 0.256281 |  | 96.6856 | 97.3857 |
| 0.261307 |  | 97.8436 | 98.282 |
| 0.266332 |  | 98.4914 | 98.9457 |
| 0.271357 |  | 98.8647 | 99.4118 |
| 0.276382 |  | 99.1444 | 99.7151 |
| 0.281407 |  | 99.3634 | 99.8907 |
| 0.286432 |  | 99.5299 | 99.9735 |
| 0.291457 |  | 99.6522 | 99.9983 |
| 0.296482 |  | 99.7383 | 100 |
| 0.301508 |  | 99.7965 | 100 |
| 0.306533 |  | 99.8351 | 100 |
| 0.311558 |  | 99.8621 | 100 |
| 0.316583 |  | 99.8847 | 100 |
| 0.321608 |  | 99.9048 | 100 |
| 0.326633 |  | 99.9223 | 100 |
| 0.33 | 39 | 99.9328 | 100 |
| 0.331658 |  | 99.9376 | 100 |
| 0.336683 |  | 99.9507 | 100 |
| 0.341709 |  | 99.9618 | 100 |
| 0.346734 |  | 99.9712 | 100 |
| 0.351759 |  | 99.9788 | 100 |
| 0.356784 |  | 99.985 | 100 |
| 0.361809 |  | 99.9898 | 100 |
| 0.366834 |  | 99.9935 | 100 |
| 0.371859 |  | 99.9962 | 100 |
| 0.376884 |  | 99.998 | 100 |
| 0.38191 |  | 99.9991 | 100 |
| 0.386935 |  | 99.9997 | 100 |
| 0.39196 |  | 99.9999 | 100 |
| 0.396985 |  | 100 | 100 |
| 0.40201 |  | 100 | 100 |
| 0.407035 |  | 100 | 100 |
| 0.41206 |  | 100 | 100 |
| 0.417085 |  | 100 | 100 |
| 0.422111 |  | 100 | 100 |
| 0.427136 |  | 100 | 100 |
| 0.432161 |  | 100 | 100 |
| 0.437186 |  | 100 | 100 |
| 0.442211 |  | 100 | 100 |
| 0.447236 |  | 100 | 100 |
| 0.452261 |  | 100 | 100 |
| 0.457286 |  | 100 | 100 |
| 0.462312 |  | 100 | 100 |
| 0.467337 |  | 100 | 100 |
| 0.472362 |  | 100 | 100 |
| 0.477387 |  | 100 | 100 |
| 0.482412 |  | 100 | 100 |
| 0.487437 |  | 100 | 100 |
| 0.492462 |  | 100 | 100 |
| 0.497487 |  | 100 | 100 |
| 0.5 | 89 | 100 | 100 |
| 0.502513 |  | 100 | 100 |
| 0.507538 |  | 100 | 100 |
| 0.512563 |  | 100 | 100 |
| 0.517588 |  | 100 | 100 |
| 0.522613 |  | 100 | 100 |
| 0.527638 |  | 100 | 100 |
| 0.532663 |  | 100 | 100 |
| 0.537688 |  | 100 | 100 |
| 0.542714 |  | 100 | 100 |
| 0.547739 |  | 100 | 100 |
| 0.552764 |  | 100 | 100 |
| 0.557789 |  | 100 | 100 |
| 0.562814 |  | 100 | 100 |
| 0.567839 |  | 100 | 100 |
| 0.572864 |  | 100 | 100 |
| 0.577889 |  | 100 | 100 |
| 0.582915 |  | 100 | 100 |
| 0.58794 |  | 100 | 100 |
| 0.592965 |  | 100 | 100 |
| 0.59799 |  | 100 | 100 |
| 0.603015 |  | 100 | 100 |
| 0.60804 |  | 100 | 100 |
| 0.613065 |  | 100 | 100 |
| 0.61809 |  | 100 | 100 |
| 0.623116 |  | 100 | 100 |
| 0.628141 |  | 100 | 100 |
| 0.633166 |  | 100 | 100 |
| 0.638191 |  | 100 | 100 |
| 0.643216 |  | 100 | 100 |
| 0.648241 |  | 100 | 100 |
| 0.653266 |  | 100 | 100 |
| 0.658291 |  | 100 | 100 |
| 0.663317 |  | 100 | 100 |
| 0.668342 |  | 100 | 100 |
| 0.67 | 97 | 100 | 100 |
| 0.673367 |  | 100 | 100 |
| 0.678392 |  | 100 | 100 |
| 0.683417 |  | 100 | 100 |
| 0.688442 |  | 100 | 100 |
| 0.693467 |  | 100 | 100 |
| 0.698492 |  | 100 | 100 |
| 0.703518 |  | 100 | 100 |
| 0.708543 |  | 100 | 100 |
| 0.713568 |  | 100 | 100 |
| 0.718593 |  | 100 | 100 |
| 0.723618 |  | 100 | 100 |
| 0.728643 |  | 100 | 100 |
| 0.733668 |  | 100 | 100 |
| 0.738693 |  | 100 | 100 |
| 0.743719 |  | 100 | 100 |
| 0.748744 |  | 100 | 100 |
| 0.75 | 97 | 100 | 100 |
| 0.753769 |  | 100 | 100 |
| 0.758794 |  | 100 | 100 |
| 0.763819 |  | 100 | 100 |
| 0.768844 |  | 100 | 100 |
| 0.773869 |  | 100 | 100 |
| 0.778894 |  | 100 | 100 |
| 0.78392 |  | 100 | 100 |
| 0.788945 |  | 100 | 100 |
| 0.79397 |  | 100 | 100 |
| 0.798995 |  | 100 | 100 |
| 0.80402 |  | 100 | 100 |
| 0.809045 |  | 100 | 100 |
| 0.81407 |  | 100 | 100 |
| 0.819095 |  | 100 | 100 |
| 0.824121 |  | 100 | 100 |
| 0.829146 |  | 100 | 100 |
| 0.834171 |  | 100 | 100 |
| 0.839196 |  | 100 | 100 |
| 0.844221 |  | 100 | 100 |
| 0.849246 |  | 100 | 100 |
| 0.854271 |  | 100 | 100 |
| 0.859296 |  | 100 | 100 |
| 0.864322 |  | 100 | 100 |
| 0.869347 |  | 100 | 100 |
| 0.874372 |  | 100 | 100 |
| 0.879397 |  | 100 | 100 |
| 0.884422 |  | 100 | 100 |
| 0.889447 |  | 100 | 100 |
| 0.894472 |  | 100 | 100 |
| 0.899497 |  | 100 | 100 |
| 0.904523 |  | 100 | 100 |
| 0.909548 |  | 100 | 100 |
| 0.914573 |  | 100 | 100 |
| 0.919598 |  | 100 | 100 |
| 0.924623 |  | 100 | 100 |
| 0.929648 |  | 100 | 100 |
| 0.934673 |  | 100 | 100 |
| 0.939698 |  | 100 | 100 |
| 0.944724 |  | 100 | 100 |
| 0.949749 |  | 100 | 100 |
| 0.954774 |  | 100 | 100 |
| 0.959799 |  | 100 | 100 |
| 0.964824 |  | 100 | 100 |
| 0.969849 |  | 100 | 100 |
| 0.974874 |  | 100 | 100 |

% Dissolved

| Time (h) | Reported | Predicted with out surface pH | Predicted with surface pH |
| --- | --- | --- | --- |
| 0 | 0 | 0 | 0 |
| 0.00502513 |  | 0 | 0 |
| 0.0100503 |  | 0 | 0 |
| 0.0150754 |  | 0 | 0 |
| 0.0201005 |  | 0 | 0 |
| 0.0251256 |  | 0 | 0 |
| 0.0301508 |  | 0 | 0 |
| 0.0351759 |  | 0 | 0 |
| 0.040201 |  | 0 | 0 |
| 0.0452261 |  | 0 | 0 |
| 0.0502513 |  | 0 | 0 |
| 0.0552764 |  | 0 | 0 |
| 0.0603015 |  | 0 | 0 |
| 0.0653266 |  | 0 | 0 |
| 0.0703518 |  | 0 | 0 |
| 0.0753769 |  | 0 | 0 |
| 0.080402 |  | 0 | 0 |
| 0.0854271 |  | 0 | 0 |
| 0.0904523 |  | 0 | 0 |
| 0.0954774 |  | 0 | 0 |
| 0.100503 |  | 0 | 0 |
| 0.105528 |  | 0 | 0 |
| 0.110553 |  | 0 | 0 |
| 0.115578 |  | 0 | 0 |
| 0.120603 |  | 0 | 0 |
| 0.125628 |  | 0 | 0 |
| 0.130653 |  | 0 | 0 |
| 0.135678 |  | 0 | 0 |
| 0.140704 |  | 0 | 0 |
| 0.145729 |  | 0 | 0 |
| 0.150754 |  | 0 | 0 |
| 0.155779 |  | 0 | 0 |
| 0.160804 |  | 0 | 0 |
| 0.165829 |  | 0 | 0 |
| 0.170854 |  | 0 | 0 |
| 0.175879 |  | 0 | 0 |
| 0.180905 |  | 0 | 0 |
| 0.18593 |  | 0 | 0 |
| 0.190955 |  | 0 | 0 |
| 0.19598 |  | 0 | 0 |
| 0.201005 |  | 0 | 0 |
| 0.20603 |  | 0 | 0 |
| 0.211055 |  | 0 | 0 |
| 0.21608 |  | 0 | 0 |
| 0.221106 |  | 0 | 0 |
| 0.226131 |  | 0 | 0 |
| 0.231156 |  | 0 | 0 |
| 0.236181 |  | 7.04811 | 18.8048 |
| 0.241206 |  | 21.3147 | 32.0799 |
| 0.246231 |  | 30.247 | 39.2677 |
| 0.25 | 22 | 36.3256 | 42.9567 |
| 0.251256 |  | 41.1908 | 44.0665 |
| 0.256281 |  | 45.2248 | 48.0753 |
| 0.261307 |  | 46.6436 | 51.4802 |
| 0.266332 |  | 51.5924 | 54.3772 |
| 0.271357 |  | 54.1724 | 56.8454 |
| 0.276382 |  | 54.1724 | 58.9513 |
| 0.281407 |  | 54.1724 | 60.7515 |
| 0.286432 |  | 54.1724 | 62.2948 |
| 0.291457 |  | 54.1724 | 63.6244 |
| 0.296482 |  | 54.1724 | 64.7786 |
| 0.301508 |  | 54.1724 | 65.7925 |
| 0.306533 |  | 54.1724 | 66.698 |
| 0.311558 |  | 54.1724 | 67.5251 |
| 0.316583 |  | 54.1724 | 68.3019 |
| 0.321608 |  | 54.1724 | 69.0497 |
| 0.326633 |  | 54.1724 | 69.7731 |
| 0.33 | 42 | 54.1724 | 70.2444 |
| 0.331658 |  | 54.1724 | 70.4727 |
| 0.336683 |  | 54.1724 | 71.1494 |
| 0.341709 |  | 54.1724 | 71.8041 |
| 0.346734 |  | 54.1724 | 72.4374 |
| 0.351759 |  | 54.1724 | 73.05 |
| 0.356784 |  | 54.1724 | 73.6428 |
| 0.361809 |  | 54.1724 | 74.2163 |
| 0.366834 |  | 54.1724 | 74.7712 |
| 0.371859 |  | 54.1724 | 75.3081 |
| 0.376884 |  | 54.1724 | 75.8276 |
| 0.38191 |  | 54.1724 | 76.3302 |
| 0.386935 |  | 54.1724 | 76.8166 |
| 0.39196 |  | 54.1724 | 77.2871 |
| 0.396985 |  | 54.1724 | 77.7424 |
| 0.40201 |  | 54.1724 | 78.1829 |
| 0.407035 |  | 54.1724 | 78.6091 |
| 0.41206 |  | 54.1724 | 79.0215 |
| 0.417085 |  | 54.1724 | 79.4204 |
| 0.422111 |  | 54.1724 | 79.8063 |
| 0.427136 |  | 54.1724 | 80.1797 |
| 0.432161 |  | 54.1724 | 80.5408 |
| 0.437186 |  | 54.1724 | 80.8901 |
| 0.442211 |  | 54.1724 | 81.2279 |
| 0.447236 |  | 54.1724 | 81.5546 |
| 0.452261 |  | 54.1724 | 81.8706 |
| 0.457286 |  | 54.1724 | 82.1762 |
| 0.462312 |  | 54.1724 | 82.4717 |
| 0.467337 |  | 54.1724 | 82.7574 |
| 0.472362 |  | 54.1724 | 83.0336 |
| 0.477387 |  | 54.1724 | 83.3006 |
| 0.482412 |  | 54.1724 | 83.5587 |
| 0.487437 |  | 54.1724 | 83.8083 |
| 0.492462 |  | 54.1724 | 84.0495 |
| 0.497487 |  | 54.1724 | 84.2827 |
| 0.5 | 72 | 54.1724 | 84.3963 |
| 0.502513 |  | 54.1724 | 84.508 |
| 0.507538 |  | 54.1724 | 84.7258 |
| 0.512563 |  | 54.1724 | 84.9363 |
| 0.517588 |  | 54.1724 | 85.1397 |
| 0.522613 |  | 54.1724 | 85.3364 |
| 0.527638 |  | 54.1724 | 85.5264 |
| 0.532663 |  | 54.1724 | 85.7101 |
| 0.537688 |  | 54.1724 | 85.8876 |
| 0.542714 |  | 54.1724 | 86.0593 |
| 0.547739 |  | 54.1724 | 86.2252 |
| 0.552764 |  | 54.1724 | 86.3856 |
| 0.557789 |  | 54.1724 | 86.5408 |
| 0.562814 |  | 54.1724 | 86.6909 |
| 0.567839 |  | 54.1724 | 86.8361 |
| 0.572864 |  | 54.1724 | 86.9766 |
| 0.577889 |  | 54.1724 | 87.1126 |
| 0.582915 |  | 54.1724 | 87.2444 |
| 0.58794 |  | 54.1724 | 87.372 |
| 0.592965 |  | 54.1724 | 87.4957 |
| 0.59799 |  | 54.1724 | 87.6156 |
| 0.603015 |  | 54.1724 | 87.732 |
| 0.60804 |  | 54.1724 | 87.845 |
| 0.613065 |  | 54.1724 | 87.9548 |
| 0.61809 |  | 54.1724 | 88.0615 |
| 0.623116 |  | 54.1724 | 88.1654 |
| 0.628141 |  | 54.1724 | 88.2666 |
| 0.633166 |  | 54.1724 | 88.3653 |
| 0.638191 |  | 54.1724 | 88.4617 |
| 0.643216 |  | 54.1724 | 88.5558 |
| 0.648241 |  | 54.1724 | 88.648 |
| 0.653266 |  | 54.1724 | 88.7382 |
| 0.658291 |  | 54.1724 | 88.8268 |
| 0.663317 |  | 54.1724 | 88.9138 |
| 0.668342 |  | 54.1724 | 88.9995 |
| 0.67 | 84 | 54.1724 | 89.0275 |
| 0.673367 |  | 54.1724 | 89.0839 |
| 0.678392 |  | 54.1724 | 89.1673 |
| 0.683417 |  | 54.1724 | 89.2497 |
| 0.688442 |  | 54.1724 | 89.3314 |
| 0.693467 |  | 54.1724 | 89.4125 |
| 0.698492 |  | 54.1724 | 89.493 |
| 0.703518 |  | 54.1724 | 89.573 |
| 0.708543 |  | 54.1724 | 89.6524 |
| 0.713568 |  | 54.1724 | 89.7313 |
| 0.718593 |  | 54.1724 | 89.8096 |
| 0.723618 |  | 54.1724 | 89.8873 |
| 0.728643 |  | 54.1724 | 89.9645 |
| 0.733668 |  | 54.1724 | 90.0412 |
| 0.738693 |  | 54.1724 | 90.1173 |
| 0.743719 |  | 54.1724 | 90.1929 |
| 0.748744 |  | 54.1724 | 90.2679 |
| 0.75 | 89 | 54.1724 | 90.2866 |
| 0.753769 |  | 54.1724 | 90.3425 |
| 0.758794 |  | 54.1724 | 90.4165 |
| 0.763819 |  | 54.1724 | 90.4899 |
| 0.768844 |  | 54.1724 | 90.5629 |
| 0.773869 |  | 54.1724 | 90.6353 |
| 0.778894 |  | 54.1724 | 90.7073 |
| 0.78392 |  | 54.1724 | 90.7787 |
| 0.788945 |  | 54.1724 | 90.8496 |
| 0.79397 |  | 54.1724 | 90.92 |
| 0.798995 |  | 54.1724 | 90.9899 |
| 0.80402 |  | 54.1724 | 91.0593 |
| 0.809045 |  | 54.1724 | 91.1283 |
| 0.81407 |  | 54.1724 | 91.1967 |
| 0.819095 |  | 54.1724 | 91.2646 |
| 0.824121 |  | 54.1724 | 91.3321 |
| 0.829146 |  | 54.1724 | 91.3991 |
| 0.834171 |  | 54.1724 | 91.4656 |
| 0.839196 |  | 54.1724 | 91.5316 |
| 0.844221 |  | 54.1724 | 91.5971 |
| 0.849246 |  | 54.1724 | 91.6622 |
| 0.854271 |  | 54.1724 | 91.7268 |
| 0.859296 |  | 54.1724 | 91.791 |
| 0.864322 |  | 54.1724 | 91.8546 |
| 0.869347 |  | 54.1724 | 91.9179 |
| 0.874372 |  | 54.1724 | 91.9806 |
| 0.879397 |  | 54.1724 | 92.043 |
| 0.884422 |  | 54.1724 | 92.1048 |
| 0.889447 |  | 54.1724 | 92.1662 |
| 0.894472 |  | 54.1724 | 92.2272 |
| 0.899497 |  | 54.1724 | 92.2877 |
| 0.904523 |  | 54.1724 | 92.3478 |
| 0.909548 |  | 54.1724 | 92.4075 |
| 0.914573 |  | 54.1724 | 92.4667 |
| 0.919598 |  | 54.1724 | 92.5255 |
| 0.924623 |  | 54.1724 | 92.5838 |
| 0.929648 |  | 54.1724 | 92.6418 |
| 0.934673 |  | 54.1724 | 92.6993 |
| 0.939698 |  | 54.1724 | 92.7564 |
| 0.944724 |  | 54.1724 | 92.813 |
| 0.949749 |  | 54.1724 | 92.8693 |
| 0.954774 |  | 54.1724 | 92.9251 |
| 0.959799 |  | 54.1724 | 92.9805 |
| 0.964824 |  | 54.1724 | 93.0355 |
| 0.969849 |  | 54.1724 | 93.0901 |
| 0.974874 |  | 54.1724 | 93.1443 |

**Figure S3:** Predicted dissolution profiles using DDD Plus for F2 formulation (with diprotic acidifying agent) were evaluated in unbuffered media: (A) 0.1 M HCl, pH 1; (B) 0.0001 M HCl, pH 4. The dashed black line represents the model prediction using bulk pH, while the solid blue corresponds the prediction using surface pH, and filled blue circles denotes the observed data

**References:**

1.	Mooney KG, Mintun MA, Himmelstein KJ, Stella VJ. Dissolution kinetics of carboxylic acids I: effect of pH under unbuffered conditions. J Pharm Sci. 1981;70(1):13–22.

2.	Mooney KG, Mintun MA, Himmelstein KJ, Stella VJ. Dissolution kinetics of carboxylic acids II: effect of buffers. J Pharm Sci. 1981;70(1):22–32.
